# Bradymonabacteria, a novel bacterial predator group with versatile survival strategies in saline environments

**DOI:** 10.1186/s40168-020-00902-0

**Published:** 2020-08-31

**Authors:** Da-Shuai Mu, Shuo Wang, Qi-Yun Liang, Zhao-Zhong Du, Renmao Tian, Yang Ouyang, Xin-Peng Wang, Aifen Zhou, Ya Gong, Guan-Jun Chen, Joy Van Nostrand, Yunfeng Yang, Jizhong Zhou, Zong-Jun Du

**Affiliations:** 1 State Key Laboratory of Microbial Technology, Institute of Microbial Technology, Shandong University, No. 72, Jimo Binhai Road, Jimo, Qingdao, 266237 China; 2 Marine College, Shandong University, Weihai, 264209 China; 3 Institute for Environmental Genomics, University of Oklahoma, Norman, Oklahoma 73019 USA; 4 State Key Joint Laboratory of Environment Simulation and Pollution Control, School of Environment, Tsinghua University, Beijing, 100084 China

**Keywords:** Bacterial predator, *Bradymonadales*, Metabolic deficiencies, Comparative genomic analysis, Biogeographic analysis

## Abstract

**Background:**

Bacterial predation is an important selective force in microbial community structure and dynamics. However, only a limited number of predatory bacteria have been reported, and their predatory strategies and evolutionary adaptations remain elusive. We recently isolated a novel group of bacterial predators, Bradymonabacteria, representative of the novel order *Bradymonadales* in δ-*Proteobacteria*. Compared with those of other bacterial predators (e.g., *Myxococcales* and *Bdellovibrionales*), the predatory and living strategies of *Bradymonadales* are still largely unknown.

**Results:**

Based on individual coculture of Bradymonabacteria with 281 prey bacteria, Bradymonabacteria preyed on diverse bacteria but had a high preference for *Bacteroidetes*. Genomic analysis of 13 recently sequenced Bradymonabacteria indicated that these bacteria had conspicuous metabolic deficiencies, but they could synthesize many polymers, such as polyphosphate and polyhydroxyalkanoates. Dual transcriptome analysis of cocultures of Bradymonabacteria and prey suggested a potential contact-dependent predation mechanism. Comparative genomic analysis with 24 other bacterial predators indicated that Bradymonabacteria had different predatory and living strategies. Furthermore, we identified *Bradymonadales* from 1552 publicly available 16S rRNA amplicon sequencing samples, indicating that *Bradymonadales* was widely distributed and highly abundant in saline environments. Phylogenetic analysis showed that there may be six subgroups in this order; each subgroup occupied a different habitat.

**Conclusions:**

Bradymonabacteria have unique living strategies that are transitional between the “obligate” and the so-called facultative predators. Thus, we propose a framework to categorize the current bacterial predators into 3 groups: (i) obligate predators (completely prey-dependent), (ii) facultative predators (facultatively prey-dependent), and (iii) opportunistic predators (prey-independent). Our findings provide an ecological and evolutionary framework for *Bradymonadales* and highlight their potential ecological roles in saline environments.

Video abstract.

## Background

Bacterial predators have been proposed as an indispensable selective force in bacterial communities [1–4]. Predation by bacteria can release nutrients [5] and affect biogeochemical cycling. In contrast to phages, bacterial predators do not need to be present in high concentrations to drive significant bacterial mortality in the environment [6, 7]. In addition, bacterial predators have higher prey-killing efficiency in low-nutrient medium than phages [8]. However, these studies have mostly been based on *Bdellovibrio* and like organisms (BALOs), and little is known of the ecological roles of other bacterial predators.

Predatory bacteria are classified into two categories, obligate or facultative predators, based on their prey-independent or prey-dependent living strategies [9]. Obligate predators include several genera collectively known as BALOs [10]. These predatory bacteria can attack their prey by penetrating the cell wall [11], dwelling in the periplasm, and then killing their host [12]. Therefore, their lifestyle depends on the presence of their prey in the natural environment, and BALOs lose viability within several hours if the prey is not available [8, 13, 14]. Facultative predators also include several genera [9], such as *Myxococcus*, *Lysobacter*, and *Herpetosiphon* [15]. These predators kill their prey by secreting antimicrobial substances into the surrounding environment [9, 16]. In general, the so-called facultative predators have been considered to be those that can be maintained as pure bacterial cultures and be free living without their prey in natural environments. However, due to the lack of additional types of predators, no assessment could be made with respect to how dependent they were on their prey. Furthermore, whether there is a transitional type between obligate and so-called facultative predators is unclear.

Bradymonabacteria are representative of the novel order *Bradymonadales*, which are phylogenetically located in the δ-*Proteobacteria* [17]. The first type species of *Bradymonadales*, *Bradymonas sediminis* FA350^T^, was isolated in 2015 [17]. To date, 9 strains within the *Bradymonadales* have been isolated and found to belong to 7 candidate novel species; these Bradymonabacteria are bacterial predators [18]. Interestingly, the phylum *Proteobacteria* contains three orders of predatory bacteria. Among them, *Myxococcales* and *Bradymonadales* belong to δ-*Proteobacteria*, while *Bdellovibrionales* were classified as *Oligoflexia* in 2017 [19]. *Myxococcales* and *Bdellovibrionales* are so-called facultative and obligate predators, respectively. Additionally, they have different distribution patterns in the environment. *Myxococcales* are mainly found in soil and sediment niches [20, 21], while *Bdellovibrionales* are aquatic. However, how *Bradymonadales* adapt to predatory lifestyles and whether they have specific living strategies or ecological importance remain largely unknown.

Here, we analyzed the predation range of *Bradymonadales* on diverse bacteria and their predatory morphological and physiological characteristics. By using comparative genomic analysis of *Bradymonadales* and other predatory bacteria, we revealed the genetic and metabolic potential of this group. To assess the diversity and frequency of occurrence of the various ribotypes of known predators (*Bradymonadales*, *Myxococcales*, and *Bdellovibrionales*) on a global scale, we surveyed published 16S rRNA gene amplicon datasets from a number of ecosystems representing a broad range of geographic locations, climatic zones, and salinities. Our study provides an ecological and evolutionary framework for *Bradymonadales* and highlights their potential ecological roles in predation.

## Results

### Bradymonabacteria are efficient predators of diverse prey bacteria

**Fig. 1:**
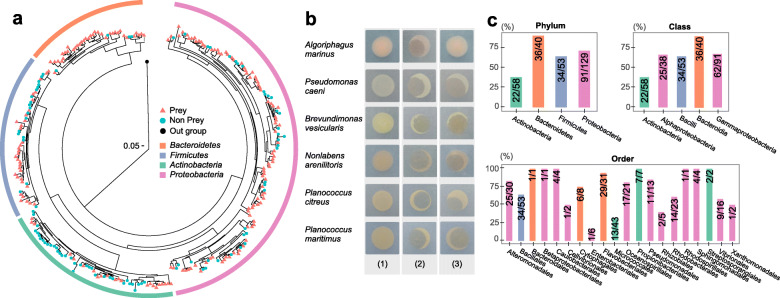
Predation assays for potential prey organisms. **a** A total of 281 organisms were selected to test predation by two type strains of *Bradymonadales*. The phylogenetic tree was analyzed for the tested organisms. Red dots on the phylogenetic tree indicate that an organism could be preyed upon by either *Bradymonas sediminis* FA350^T^ or *Lujinxingia litoralis* B210^T^. Green dots indicate that the organism could not be preyed upon by either tested predator. Detailed information about the organisms on the tree is shown in Table S1. **b** The predation phenotype of *Bradymonas sediminis* FA350^T^ or *Lujinxingia litoralis* B210^T^ on prey. The numbers 1 to 3 indicate pure culture of different prey, mixed culture of *Bradymonas sediminis* FA350^T^ and prey, and mixed culture of *Lujinxingia litoralis* B210^T^ and prey, respectively. **c** The percentage of organisms that could be preyed upon is shown in the bar chart

In total, 9 strains of bacteria in the novel order *Bradymonadales* were isolated using the enrichment culture method [22]. Among these strains, eight strains were isolated from costal sediment sampled in Weihai, China, while strain YN101 was isolated from a Gaodao saltern (36° 54′ N, 122° 14′ E) in Weihai, China. Strains FA350^T^ [17, 18] and B210^T^ [23] are the two type strains for different genera of *Bradymonadales*. Both these type strains were used to investigate the predator-prey range of Bradymonabacteria. A total of 281 isolated bacteria were cocultured with Bradymonabacteria FA350^T^ [17, 18] or B210^T^ [23] as lawns in individual Petri dishes (Fig. 1a, Table S1). Zones of predation were measured (Fig. 1b), and the results showed that the Bradymonabacteria preyed on diverse bacteria but showed a strong preference for *Bacteroidetes* (90% of tested bacteria could be preyed on) and *Proteobacteria* (71% of tested bacteria could be preyed on) (Fig. 1c). Predation on bacteria in the orders *Flavobacteriales*, *Caulobacterales*, *Propionibacteriales*, and *Pseudomonadales* was broadly distributed, with a mean predation percentage greater than 90%, while predation of *Micrococcales* and *Enterobacteriales* was less efficient.

**Fig. 2:**
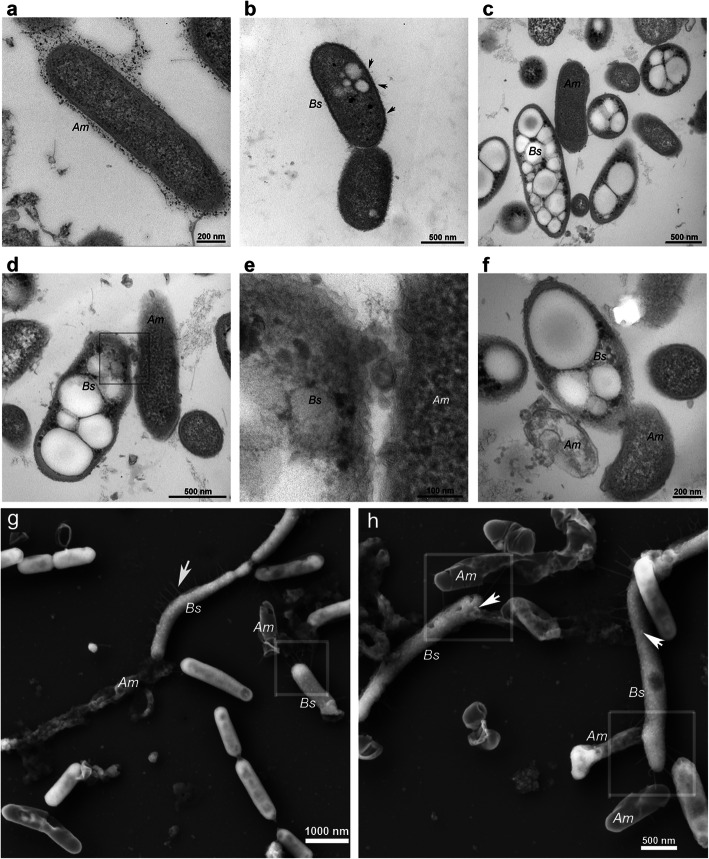
TEM and SEM micrographs of *Bradymonas sediminis* FA350^T^ (predator) and *Algoriphagus marinus* am2 (prey). We selected a prey, *Algoriphagus marinus* am2, which was smaller than the predator FA350^T^. **a** The free-living prey *Algoriphagus marinus* am2 (*Am*) in pure culture. Bar = 200 nm. **b** The free-living predator *Bradymonas sediminis* FA350^T^ (*Bs*) in pure culture. The white globose granules in the *Bs* cell indicate PHA accumulation, and the black arrows indicate type IV pili. Bar = 500 nm. **c** *Bradymonas sediminis* FA350^T^ (*Bs*) cells cocultured with *Algoriphagus marinus* am2 (*Am*) prey cells. The white globose granules in the cell indicate PHA accumulation, and the electron-dense (black) intracellular granules indicate polyphosphate. Bar = 500 nm. **d** *Bradymonas sediminis* FA350^T^ (*Bs*) cell attached to an *Algoriphagus marinus* am2 (*Am*) prey cell with OMV-like structures (shown in box area). Bar = 500 nm. **e** Enlargement of the boxed area in figure (**d**). Bar = 100 nm. **f** *Bradymonas sediminis* FA350^T^ (*Bs*) cell attached to an emptied and dead *Algoriphagus marinus* am2 (*Am*) prey cell. Bar = 200 nm. **g** SEM analysis of *Bradymonas sediminis* FA350^T^ (*Bs*) cells cocultured with *Algoriphagus marines* am2 (*Am*) prey cells. The white arrow indicates type IV pili, and the boxed area indicates *Bs* contact with the emptied *Am* with type IV pili. Bar = 1000 nm. **h** SEM analysis of *Bradymonas sediminis* FA350^T^ (*Bs*) cells attached to an *Algoriphagus marinus* am2 (*Am*) prey cell with type IV pili (shown in boxed area). The white arrows indicate the OMV-like structures. Bar = 500 nm

Transmission electron microscopy (TEM) and scanning electron microscopy (SEM) analyses were performed to understand the mechanism of predation of strain FA350^T^ on the subcellular level. Lysis of the prey cells (Fig. 2a) was detected near strain FA350^T^ in both the TEM and SEM analyses (Fig. 2). Strain FA350^T^ was found to have pili (Fig. 2b, g) and outer membrane vesicle (OMV)-like structures (Fig. 2d, e, f, h). In addition, FA350^T^ cells contained intracellular particles with low electron density (Fig. 2b, c, d, f), which were shown to contain polyhydroxyalkanoates (PHAs) by Nile blue A staining. FA350^T^ cells also contained several electron-dense (black) spots (Fig. 2b, c, d, f), which indicated the presence of intracellular polyphosphate granules [24]. Both of these particle types significantly accumulated during predation (Fig. 2).

### Bradymonabacteria are polyauxotrophs

To explore the metabolic capabilities and predation mechanism of this novel group, we analyzed 13 genomes of *Bradymonadales* (9 high-quality genomes sequenced from cultured strains and 4 reconstructed from published studies [25]). The genome size of Bradymonabacteria ranged from 5.0 to 8.0 Mb (Fig. S1a). Average nucleotide identity (ANI) analysis of the 9 cultured strains of *Bradymonadales* revealed 7 different species [26] (Fig. S1b). Other general features of the genomes are described in the Supplementary Materials (Supplementary Materials Results and Fig. S1a).

**Fig. 3:**
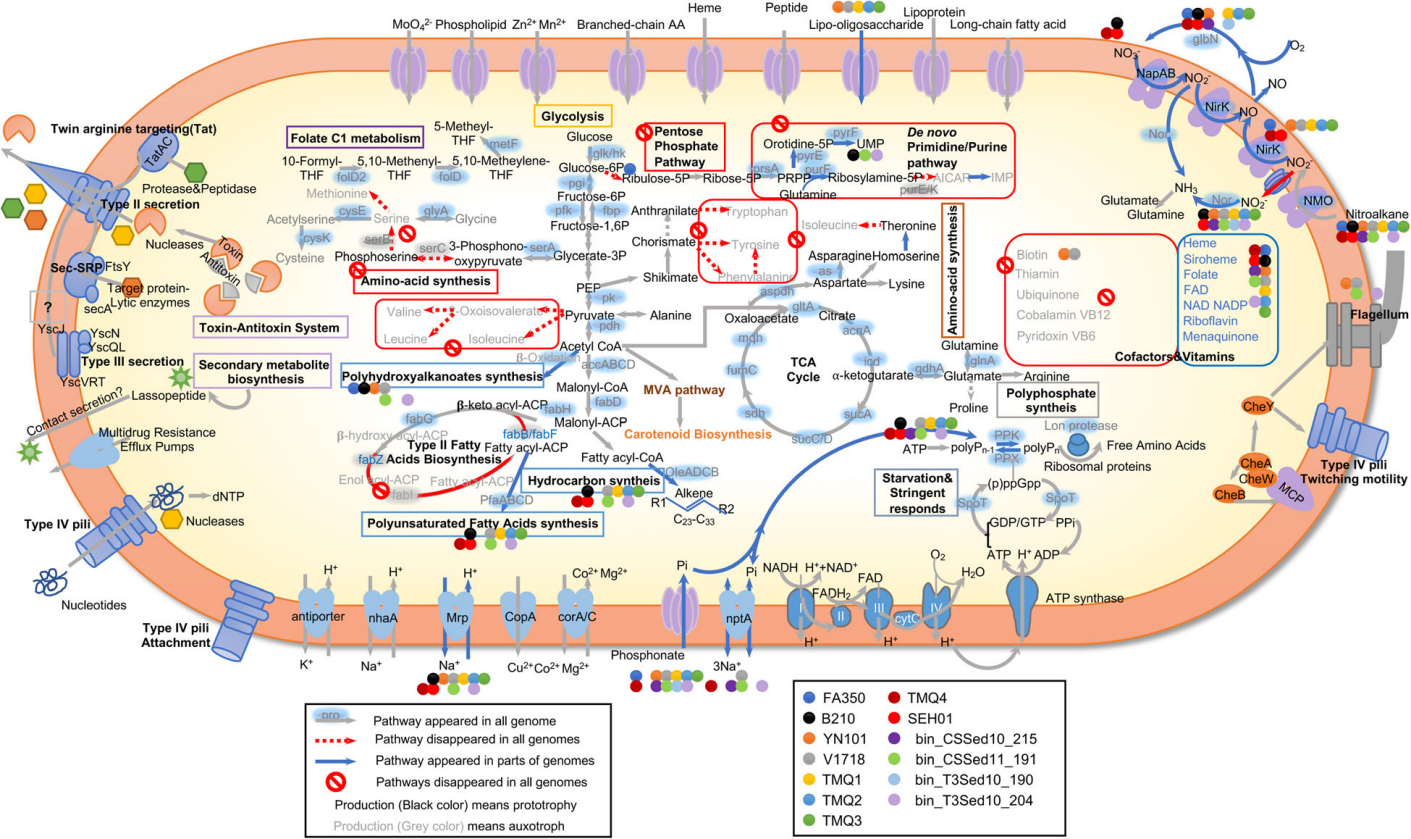
Metabolic capabilities of Bradymonabacteria. Metabolic predictions were mainly generated by referring to the KEGG and SEED database interface. Each subgroup of Bradymonabacteria is depicted as a colored circle (see figure legend). Functional genes (abbreviation according to KEGG) encoding the relevant proteins/enzymes are labeled for each metabolic step where colored circles (that is, Bradymonabacteria strains) are depicted to show the potential functions of each subgroup if any. The gray arrows indicate the corresponding genes detected for the pathways in almost all the genomes, while the red arrows indicate the corresponding genes missing from the pathways. The red “no entry” signs indicate the many key genes in pathways that are missing. All putative transporters and F0F1 ATPases are shown as well as secretion systems, type IV pili, and predicted components of flagella. The process of starvation and stringent-responsive system remodeling is mediated by the production of the alarmones guanosine pentaphosphate, pppGpp, and guanosine tetraphosphate, ppGpp. The key metabolic predictions are supported by the gene information in Table S2

Almost all strains (except FA350^T^) possessed a minimal pentose phosphate pathway, which lacked key steps for the synthesis of ribose 5-phosphate (Fig. 3, Table S2) [27]. Most of the bradymonabacterial genomes lacked key enzymes for pyrimidine synthesis, such as aspartate carbamoyltransferase, which catalyzes the first step in the pyrimidine biosynthetic pathway. All genomes lacked the complete purine de novo pathway; they were missing the phosphoribosylaminoimidazole carboxylase catalytic subunit or even the whole pathway.

In addition to this auxotrophy in the synthesis of pentose and nucleotides, all the genomes lacked complete pathways for the synthesis of many amino acids, such as serine, methionine, valine, leucine, isoleucine, histidine, tryptophan, tyrosine, and phenylalanine (Fig. 3). For example, all the genomes encoded a potential D-3-phosphoglycerate dehydrogenase for the conversion of glycerate-3P into 3-phosphonooxypyruvate for amino-acid synthesis (Fig. 3). However, in all members of Bradymonabacteria, this pathway appeared to be blocked at the subsequent step because of the absence of phosphoserine aminotransferase, although Bradymonabacteria could continue with subsequent pathways to complete the biosynthesis of cysteine and glycine. Additionally, many cofactors and vitamins that promote bacterial growth [22], such as biotin, thiamin, ubiquinone, VB_12_, and VB_6_, could not be synthesized by the de novo pathway in almost all the genomes. Notably, all the genomes had an incomplete pathway for type II fatty acid biosynthesis, lacking the key enzymes 3-oxoacyl-[acyl-carrier-protein] synthase I/II (FabB/F) and enoyl-[acyl-carrier-protein] reductase (FabI/L).

### Dual transcriptome analysis of the potential predation mechanism of Bradymonabacteria

To further determine the genes involved in predation, we performed dual transcriptome analysis of *Bradymonas sediminis* FA350^T^ with and without preying on *Algoriphagus marinus* am2 (Fig. S2). As with obligate predators, one way that Bradymonabacteria kill their prey bacteria is likely by using contact-dependent mechanisms. Here, the bradymonabacterial genomes possessed complete type IV pili (T4P) (Fig. 3), and the attached areas showed more type IV pili than the unattached areas (SEM, Fig. 2g, h). The dual-transcriptome analysis showed that genes encoding the type IV pili twitching motility protein PilT (DN745_17255) were significantly upregulated during predation (Fig. S3), suggesting that these genes may be involved in predation. Bradymonabacteria also had T4b pilins showing homology to those in *Bdellovibrio bacteriovorus* HD100, in which T4b pilins are necessary for predation [28, 29] (Fig. S4), so T4b pilins may also participate in regulating predation in Bradymonabacteria. In addition, this group of bacteria had type II and type III secretion systems (the YscRSTUV proteins that form a membrane-embedded complex known as the “export apparatus” [30]). The dual-transcriptome analysis also supported the prediction that genes encoding the type III secretion system inner-membrane protein complex (DN745_01900, DN745_10315, DN745_17280, DN745_03325, and DN745_00480) were significantly upregulated during predation (Fig. S3), implying that these genes may also be involved in predation.

Another way that Bradymonabacteria kill their prey bacteria is likely by secreting antimicrobial substances into the surrounding environment. As in most facultative bacterial predators, a few potential antimicrobial clusters for secondary metabolite synthesis, such as Lasso peptide [31], were identified in almost all genomes of Bradymonabacteria (Fig. 3). Genes involved in outer membrane vesicle (OMV) biosynthesis were also detected in most genomes, such as *ompA* (cell envelope biogenesis protein), *envC* (Murein hydrolase activator), and *tolR* (envelope stability) [32]. Vesicle membrane-related genes (DN745_03865, DN745_02930, and DN745_07125) were significantly upregulated during predation (Table S4, Fig. S3). However, the fermentation supernatant of Bradymonabacteria showed no antibacterial activity.

### Bradymonabacteria are novel predators different from the so-called obligate or facultative predators

**Fig. 4:**
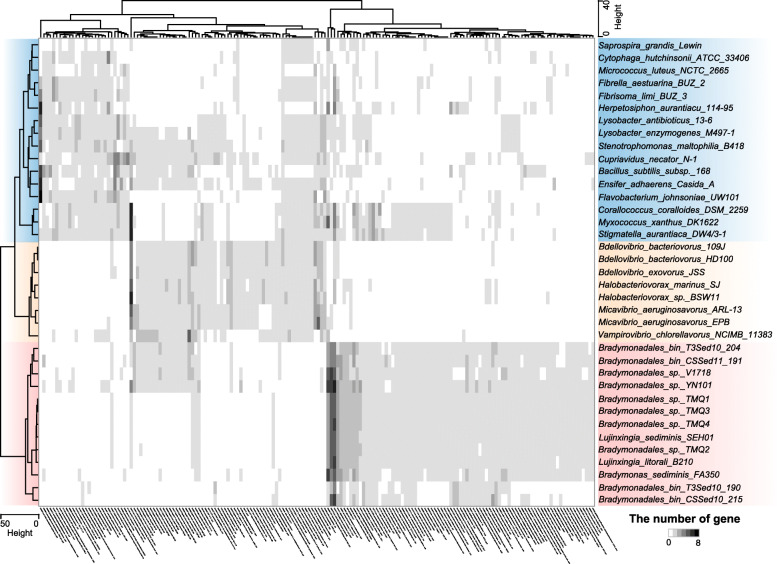
Gene abundance in facultative and obligate bacterial predators. The heatmap is based on a two-way cluster analysis of the genomic abundance of genes encoding KEGG protein groups specific to either facultative predators or obligate predators. Groups with a blue background indicate the so-called facultative predators, groups with a yellow background indicate the so-called obligate predators, and groups with a red background indicate Bradymonabacteria. The two-way cluster analysis was clustered using the ward.D2 method based on Euclidean distances. The gene abundance matrix is available in Table S3

Comparative genomic analysis with other bacterial predators was performed to explore whether Bradymonabacteria have a unique living strategy. Two-way cluster analysis showed that bradymonabacterial genomes contained features different from those of either obligate or facultative predators, which were phylogenetically located in a different branch (Fig. 4). The specific multiple metabolic deficiencies of Bradymonabacteria had some similarities to those of most obligate predators. For example, both Bradymonabacteria and obligate predators possessed a minimal pentose phosphate pathway, lacked key enzymes for pyrimidine synthesis, and lacked complete pathways for the synthesis of many amino acids, cofactors, and vitamins (Fig. 4). However, Bradymonabacteria with multiple auxotrophies could grow on common media (such as marine agar medium), though at a low growth rate [33], unlike obligate predators.

Unlike most obligate predators, the polyphosphate accumulation pathway, containing a pair of genes (polyphosphate kinase and exopolyphosphatase) associated with both polyphosphate formation and degradation [34], was present in most Bradymonabacteria (Fig. 4). Polyphosphate accumulation was also detected in FA350^T^ cells during predation (Fig. 2). In contrast to most of the other predator genomes, potential PHA synthesis from β-oxidation of fatty acids [35] was observed in most bradymonabacterial genomes (Fig. 3). In the present study, TEM analysis showed that strain FA350^T^ could significantly accumulate PHAs during predation observed in co-culture with pure culture (Fig. 2). Despite their incomplete fatty acid biosynthetic pathway, all Bradymonabacteria had a high copy number of long-chain fatty acid transporters (fadL) compared to those of other predators, allowing them to gather fatty acids from the environment (Fig. 4). In addition, genes associated with alkane synthesis, which is important for maintaining cell membrane integrity and adapting to cold environments [36], were present in most genomes of Bradymonabacteria (Figs. 3 and 4). Thus, we proposed that Bradymonabacteria could be categorized as novel predators different from so-called obligate or facultative predators (Table 1).

**Table 1:** The features of 3 different types of bacterial predators

Current predator type	Redefined predator type	Prey dependent/independent	Metabolic pathway deficiencies	Pure-culture cultivable	Storing nutrients as polymers	Predation strategy	Predation specificity
Obligate	Obligate	Completely prey-dependent	High	Extremely difficult	None	Contact-dependent	Gram-negative
Bradymonabacteria	Facultative	Facultatively prey-dependent	High	Difficult	Polyhydroxyalkanoates, polyphosphate, and alkanes	Contact-dependent	Gram-negative and Gram-positive
Facultative	Opportunistic	Prey-independent	Low	Normal	Polyphosphate^a^	Mostly contact-independent	Gram-negative and Gram-positive

^a^A polyphosphate accumulation pathway was found in genomes but not determined by experiments

### *Bradymonadales* are mainly distributed in saline environments with high diversity

**Fig. 5:**
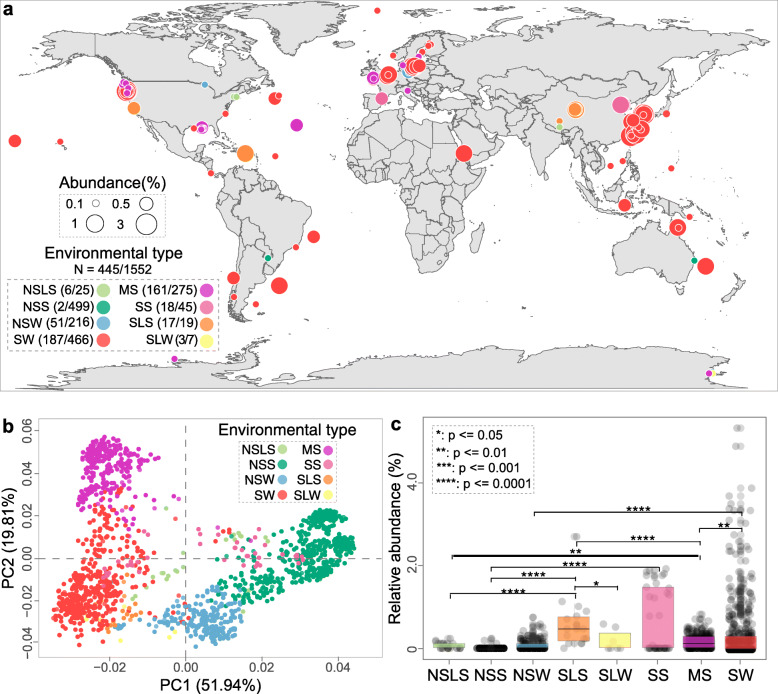
Global distribution and biodiversity patterns of Bradymonabacteria in eight types of biotopes from 1552 samples. **a** Global abundance of Bradymonabacteria. The abundance of 16S rRNA gene sequences of Bradymonabacteria is shown relative to the total prokaryotic sequences in the selected samples. Each node represents one sample. The node color indicates the type of biotope, and the node size represents the relative abundance in the corresponding samples. The bold numbers represent the number of samples where Bradymonabacteria were detected. **b** Beta diversity among all the biologically independent samples: principal component analysis (PCA) of the Bray-Curtis dissimilarity matrix, PC1 versus PC2. The clustering of all samples could be mainly explained by the type of biotopes. **c** Relative abundance of 16S rRNA gene sequences among eight types of habitats. This relative abundance of Bradymonabacteria sequences was computed within each habitat (Table S5), and the significant differences among the different biotopes were assessed by the Kruskal−Wallis test. Abbreviations: NSLS, nonsaline lake sediments; NSS, nonsaline soil; NSW, nonsaline water; SLS, saline lake sediments; SLW, saline lake water; SS, saline soil; MS, marine sediments; SW, sea water

To evaluate the global prevalence of the *Bradymonadales* order, we surveyed recently published 16S rRNA gene amplicon studies that provided high taxonomic resolution along with relative sequence abundances. The 16S rRNA gene amplicons from 1552 samples were grouped into eight types of environments (Fig. 5a and Table S5). A total of 811 samples were from inland environments, while others were from marine environments, with each biotope showing a somewhat different microbial community (Fig. 5b). Based on the alpha diversity analysis, marine sediment and soil biotopes harbored more OTUs compared with other biotopes (Fig. S5). Bradymonabacteria was detected in 348 of 741 marine samples (relative abundance > 0.01%) but only 20 of 544 soil samples (Fig. 5a). All samples were sorted into an ordination diagram based on the similarity of communities (Fig. 5b). Saline biotopes were clearly separated from nonsaline biotopes (Fig. S6), suggesting that salinity was a significant factor in shaping microbial communities. For each biotope, the relative abundance of *Bradymonadales* in the saline environments (i.e*.*, seawater and saline lake sediment) was significantly higher than that in the nonsaline environments (i.e., nonsaline soil and nonsaline water) (*P* ≤ 0.0001, Fig. 5c). The distribution analysis was consistent with the genomic feature analysis (Fig. 2), in which several genes encoding sodium symporters and Na^+^/H^+^ antiporters were found in the genomes, suggesting a beneficial effect of salinity on Bradymonabacteria.

In addition, we compared the relative abundance of *Bradymonadales* with those of two orders of well-known predatory bacteria, *Bdellovibrionales* and *Myxococcales* [12, 37, 38]. We found that *Myxococcales* and *Bdellovibrionales* were also globally distributed (Fig. S7); however, *Myxococcales* were more commonly distributed in soil and sediment environments, while *Bdellovibrionales* were more likely to be found in freshwater and seawater (Fig. S7). The total relative abundances of *Bradymonadales*, *Bdellovibrionales*, and *Myxococcales* ranged from 0.7 to 6.4% of the total prokaryotic microbes in all 1552 samples (Fig. S8a). The mean relative abundance of *Bradymonadales* (0.5%) was similar to that of *Bdellovibrionales* (0.6%) when both were detected in environmental samples (Fig. S8b). In contrast, *Bradymonadales* was one of the most abundant known predatory bacteria in saline lake sediment and saline lake water (Fig. S8c).

To further determine how salinity affected the relative abundance of *Bradymonadales*, we used the Gaodao multipond salterns as a model and applied 16S rRNA gene amplicon, fluorescence in situ hybridization (FISH) (Tables S6 and S7), and real-time PCR analyses (Figs. S8d and S9). The results showed that *Bradymonadales* appeared in all the tested multipond saltern datasets, accounting for an average of 0.74% of all bacterial sequences and more than 1.0% relative abundance within the range of 80 and 265 g/L salinity (Fig. S8d), significantly higher than those of *Bdellovibrionales* and *Myxococcales*. *Bradymonadales* may exhibit different correlations with prey at different abundances, a possibility that will require further study. In addition, fluorescence in situ hybridization (FISH) (Tables S6 and S7) and real-time PCR experiments were performed, and the results showed a relative cell abundance of *Bradymonadales* of up to 0.6% and a gene copy numbers ratio as high as 1.96% in sediments with salinity 80 g/L (Fig. S9). These findings support those of the global analysis (Fig. S8c) and suggest that *Bradymonadales* may be a dominant bacterial predator in some specific saline environments.

**Fig. 6:**
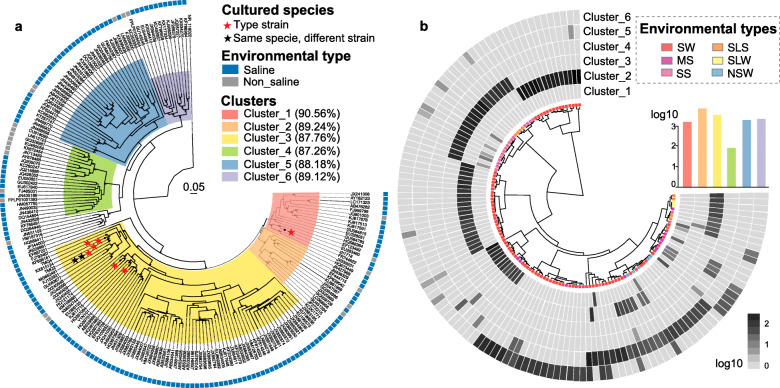
Phylogeny of 6 proposed subgroups of Bradymonabacteria. **a** Maximum likelihood phylogenetic tree of Bradymonabacteria based on 187 representative 16S rRNA gene sequences (> 1200 bp) dereplicated at a 98.5% cutoff. The subgroups from Cluster_1 to Cluster_6 were colored in the corresponding leaves of the tree and are shown with the similarity of each subgroup. The outer colored square indicates the sequence of the original biotope: nonsaline (gray) and saline (blue). Pentagram represents cultured Bradymonabacteria in our laboratory. All 16S rRNA gene sequences of Bradymonabacteria and the RAxML phylogenetic tree are available in Table S6. **b** The coverage of each subgroup of Bradymonabacteria for 127 samples. The abundance of Bradymonabacteria is expressed relative to the total number of prokaryotic sequences in the corresponding samples. The biotope types are shown by the colored nodes located under each leaf of the cluster. The bar graph indicates the read number of each cluster in the 127 samples

To explore the diversity and distinct evolution of bradymonabacterial subgroups in different biotopes, we performed a phylogenetic analysis of nearly full-length 16S rRNA gene sequences of diverse origin by maximum likelihood inference (Table S6). A total of 187 OTUs were detected and found to form six sequence clusters (Fig. 6a). Almost 87.2% of the representative sequences originated from saline biotopes (such as seawater, marine sediments, salterns, corals, and saline lakes). Since bradymonabacterial subgroups may be selectively distributed in local biotopes, we investigated the relative abundance of each subgroup throughout the 127 representative samples in which the relative abundance of *Bradymonadales* was above 1% of total 16S rRNA gene reads (Fig. 6b). Five of the 6 bradymonabacterial subgroups showed significantly higher abundance in saline environments. Cluster-2 and cluster-6 were mainly observed in seawater biotopes, whereas cluster-3 was mainly observed in marine sediment and saline lake sediment (Fig. 6b), consistent with the environments of the cultured strains. Cluster-1 and cluster-4 were both detected in marine sediment and seawater biotopes. Cluster-5 lineages tended to occur in both freshwater and seawater biotopes (Fig. 6b).

## Discussion

In all ecosystems, predation is an important interaction among living organisms. Bacterial predators are proposed to play an important role in controlling and shaping bacterial populations in diverse environments [3, 39]. However, despite their ecological importance, only a few examples of predatory bacteria have been studied in depth. Recently, many predatory bacteria from various phyla have been isolated from different environments; however, most of their predatory lifestyle strategies and adaptations remain unclear. This study systematically analyzed the predatory lifestyle adaptations, global distribution, and diversity of *Bradymonadales*; highlighted the ecological role of *Bradymonadales*; and provided a framework for the categorization of the known predatory bacteria.

In our study, based on comparative genomic and physiological analyses, Bradymonabacteria were identified as a novel group of bacterial predators with versatile survival strategies that are transitional types between “obligate” predators and the so-called facultative predators (Table 1). Similar to the obligate predators, Bradymonabacteria have multiple metabolic deficiencies. Their incomplete pathways might be important for prey-dependent growth, as the precursor compounds could be acquired from predation. In addition, the loss of genes in the fatty acid biosynthetic pathway was notable, because fatty acids are integral components of the cellular membrane, and their synthesis is considered to be a housekeeping function of cells [40]. Thus, these organisms may incorporate exogenous fatty acids from prey bacteria into their membrane phospholipids using their high copy number of long-chain fatty acid transport proteins [41] (Fig. 3). Unlike the currently called “facultative” predators, the multiple gene loss observed in Bradymonabacteria may render them more dependent on prey for their lost metabolic functions and could also provide a selective advantage by conserving predators’ limited resources [42]. However, the sequenced genomes of Bradymonabacteria were surprisingly large (5.0 to 8.0 Mb, Fig. S1a), suggesting that Bradymonabacteria are far from obligate parasites, with seemingly none of the reductive evolution that results from a parasitic lifestyle in bacteria such as *Mycobacterium leprae* [43]. The large size of their genomes may be indicative of the vast range of genes required for Bradymonabacteria to both effectively tolerate the absence of prey and carry out predation.

In contrast to most predators, Bradymonabacteria can synthesize many nutrient polymers, such as polyphosphate, PHA, and alkane molecules. Exopolyphosphatase catalyzes the hydrolysis of terminal phosphate residues from polyphosphate chains, accompanying the production of ATP and thus playing a role in the production of energy [44]. *Bradymonadales* cells may accumulate polyphosphate in the phosphate-rich zone, using it as an energy source [45]. Meanwhile, PHA granules are synthesized as sinks of excess carbon and are used as carbon and energy reserves in starvation conditions [46]. Under nutrient starvation, maintenance energy and free amino acids can be provided by endogenous substrates such as PHAs and polyphosphate [47, 48]; this ability may be an important feature for the survival of Bradymonabacteria during intervals without predation. This feature is interesting among bacterial predators, as it is commonly found in animal predation. For example, the bear can store fat in its body to ensure that it will survive the long winter. In addition, Bradymonabacteria may also synthesize alkanes to maintain cell membrane integrity [36] and complement its poor fatty acid synthesis ability. Thus, these multiple auxotrophies and ability to synthesize nutrient polymers confer on Bradymonabacteria a versatile survival strategy for natural environments, which contrasts with that of the currently known “obligate” or “facultative” predators.

As bacterial predators, Bradymonabacteria have developed a wide range of mechanisms to attack their prey. Although genes involved in OMV-like biosynthesis were detected in most genomes, the fermentation supernatant of Bradymonabacteria showed no antibacterial activity, suggesting that bradymonabacterial predation depends on the cell contact, which is different from that of the currently known facultative predators. Contact-dependent predation mechanisms allow predators to attach to the prey and then carry out predation. This prey-dependent process has a relatively low energy cost and could prevent secretory virulence factors from being diluted by the surrounding environment [49]. Bradymonabacteria also has T4P, which could pull adherent bacteria into close association with other bacteria [50]. T4P could also transport bound substrates such as DNA [51] into the periplasm and export exoproteins across the outer membrane [52]. Contact-dependent type III secretion systems have also been found in Bradymonabacteria and are reported to be capable of moving virulence factors across bacterial outer membranes and directly across the host cell membrane into the cytoplasm of a host cell [53]. However, no reports have indicated that the type III secretion system is involved in direct combat between bacteria. Whether type III secretory complexes could penetrate the bacterial cell wall is unknown. Further gene knockout experiments and systematic TEM analysis should be performed to identify whether and how the type III secretion system works during predation.

Our biogeographic analysis suggested that Bradymonabacteria are mainly distributed in saline environments, and some other studies have also detected *Bradymonadales* in hypersaline soda lake sediments [25], suggesting that saline environments could be enriched in these bacteria. Our genome analysis also showed that *Bradymonadales* had many genes encoding sodium symporters and Na^+^/H^+^ antiporters to maintain osmotic pressure in saline environments. These findings supported the global analysis (Figs. 5 and S8c), suggesting that *Bradymonadales* might be a dominant bacterial predator in some specific saline environments compared with *Bdellovibrionales* and *Myxococcales*. The analysis of the complex intragroup phylogeny of the 6 subgroups of Bradymonabacteria revealed that distinct evolutionary bradymonabacterial subgroups had arisen in different biotopes, suggesting the occurrence of adaptive evolution specific to each habitat. Patterns related to salinity status also suggest that most *Bradymonadales* are halophiles [17].

Bradymonabacteria had a very high predation efficiency on bacteria within the phylum *Bacteroidetes*. Members of the phylum *Bacteroidetes* are one the most abundant groups of bacteria in the ocean [54]. Thus, a high predation efficiency on *Bacteroidetes* may indicate that Bradymonabacteria has important roles in regulating *Bacteroidetes* communities in oceans. Furthermore, Bradymonabacteria had a high predation efficiency on *Flavobacteria* and *Proteobacteria*, some of which are commensal bacteria in fish [55], suggesting that Bradymonabacteria may be involved in fish microbiome dysbiosis. In addition, *Bradymonadales* were detected in coral samples, as Bradymonabacteria have a wide range of prey, including the coral pathogen *Vibrio harveyi*, suggesting that Bradymonabacteria may protect coral hosts by consuming potential pathogens [39]. The exact ecological roles of this group in different environments should be determined in further studies.

## Conclusion

The unique metabolic pathways of Bradymonabacteria, which include conspicuous metabolic deficiencies similar to those of obligate predators but with a more effective starvation stress response mechanism, provide these bacteria with transitional survival models between “obligate” and so-called facultative predators. We suggest that Bradymonabacteria, as facultative prey-dependent predators, can be renamed as facultative predators. In addition, the currently used “facultative” predators term can be replaced by opportunistic predators, which are prey-independent predators. Thus, we propose a framework to categorize the current bacterial predators into 3 groups: (i) obligate predators (completely prey-dependent), such as most of the BALOs; (ii) facultative predators (facultatively prey-dependent), such as the Bradymonabacteria cultured in the present study; and (iii) opportunistic predators (prey-independent), such as *Myxobacteria* and *Lysobacter* sp. (Table 1). This categorization replaces the currently known “facultative” predators with opportunistic predators and will be helpful for further study of the different ecological importance of each type of bacterial predator. The evolution of bacterial predation in these three groups of predators should also be studied in the future to better understand the significance of predation to biological evolution.

Our study highlights the ecological role of *Bradymonadales* in saline environments. Given their substantial sequence and cell frequencies in the saline environment and their storage of nutrients as polymers in cells during predation, *Bradymonadales* may have an alternative way of regulating global nutrient cycling. To better understand the impact of bradymonabacterial predation on regulating biogeochemical cycling, predation mutants and microcosms need to be developed in further studies.

## Methods

### Predation experiments

To explore the predation of Bradymonabacteria, we used *Bradymonas sediminis* FA350^T^ and *Lujinxingia litoralis* B210^T^ as representative strains. All candidate prey strains were obtained from our laboratory. Cells were centrifuged, washed, and concentrated in seawater to a final OD_600_ of 3.0 for predator strains and 6.0 for candidate prey strains. Drops of 5.0 μl of the predator strain suspensions were deposited on the surfaces of agar plates and allowed to dry. Next, 20.0 μl drops of each different candidate prey strain suspension were placed near the predator spot. The plates were incubated at 33 °C, and images were taken after 48 h with a digital camera. To detect PHA accumulation, the granules were stained with the Nile red component of Nile blue A.

### Genome sequencing and comparative genome analyses

To explore the potential metabolic capacity of bacterial predators, we sequenced 3 complete genomes and 6 draft genomes of all currently known Bradymonabacteria isolate strains. The genomic DNA of all strains was extracted with a DNA extraction kit (TaKaRa Bio) according to the manufacturer’s instructions. For strains FA350^T^, V1718, and YN101, complete genome sequencing was performed by Nanjing CocoBio Co., China, using the Illumina HiSeq platform accompanied with the SMRT platform to build Illumina PE and Pacbio libraries. The single-molecule sequencing data assembly was accomplished using SOAPdenovo v2.04 and Celera Assembler v8.0, and the results were rectified through BLAST searches in the BlastR database. The de novo assembly of the scaffolds was performed using Celera Assembler 8.0, which were then overlapped and trimmed using GapCloser v1.12 (SOAPdenovo-related software) [18]. Draft genome sequencing of the other 6 strains was performed by Shanghai Personal Biotechnology Co., Ltd. (Shanghai, China) using Solexa paired-end sequencing technology [2]. A library with an average fragment length of 400 bp was constructed, and the final genomes were assembled using SOAPdenovo version 2.04 [56]. We also retrieved 37 predator genomes from NCBI (including 4 metagenome-assembled genomes). tRNA and gene prediction were performed using tRNAscan and prodigal, respectively. The genome-based metabolic potential of the bacterial predators was predicted by BlastKOALA (https://www.kegg.jp/blastkoala/). The average nucleic acid identities among the 9 cultured Bradymonabacteria strains were calculated using pyani (https://github.com/widdowquinn/pyani), and the percentage of conserved proteins (POCP) in each strain was calculated as described previously by Qin et al. [57].

### Electron microscopy analyses

We selected *Algoriphagus marinus* am2, which is smaller than the predator *Bradymonas sediminis* FA350^T^, as prey. *Bradymonas sediminis* FA350^T^ and *Algoriphagus marinus* am2 were cultured separately to the exponential growth phase, adjusted to the same OD value, mixed together, and cocultured on marine agar medium at 33 °C for 68 h.

For TEM analysis, mixed culture samples were supported on carbon/formvar-coated copper grids. The grids were inverted over a drop of 1% uranyl acetate. Thin sections were prepared with the predator-prey cocultures at 68 h incubation. The samples were mixed with 0.5 ml of 2% glutaraldehyde in 0.1 M sodium cacodylate buffer, centrifuged and resuspended in 1 ml of the same solution for 3 h. The cells were washed in cacodylate buffer, fixed with 1% osmium tetroxide, and encased in agar. The agar-encased cells were then fixed in 2% uranyl acetate, dehydrated through an ethanol series, and embedded in Epon resin. Thin sections were cut and stained with uranyl acetate and lead citrate. Specimens were examined with a JEM-1200EX electron microscope operated at 80 kV.

For SEM analysis, mixed culture samples were washed 3 times with PBS and fixed for 1 h in 2.5% glutaraldehyde in sodium cacodylate buffer (0.1 M, pH 7.2). To dehydrate the bacteria, the EM grids underwent a series of washes in increasing concentrations of ethanol (25, 50, 75, and 96%) and placed in a vacuum overnight. The samples were coated with gold and observed using a Nova NanoSEM 450.

### Dual transcriptomic analyses

To determine the gene expression profiles of the type strain FA350^T^, *Algoriphagus marius* am2^T^ was used as prey due to its cell morphology being different from *B. sediminis* FA350^T^. Moreover, *B. sediminis* FA350^T^ predated *A. marius* am2^T^ well, and the two species have a distant evolutionary relationship that was helpful for further gene mapping analysis. A pure culture of FA350^T^ and a coculture of FA350^T^ with the prey *A. marinus* am2 were cultured on marine agar medium at 33 °C for 0 h, 68 h, and 120 h, respectively. Mixed-culture cells sedimented by centrifugation and washed, and the resulting pellets stored at − 80 °C prior to RNA extraction. Each time point was collected in triplicate (*n* = 3) for further transcriptomic analysis. RNA was extracted using an miRNEasy mini kit (Qiagen 217004), rRNA was removed using a Ribo-Zero Magnetic kit (Bacteria) from Epicentre (MRZB12424), and cDNA library construction was performed with a TruSeq Stranded mRNA library preparation kit from Illumina (RS-122-2101) [58]. Sequencing was carried out on a HiSeq sequencer at Novogene Co., Ltd. (Beijing, China).

### Transcriptome mapping and differential expression analysis

For transcriptomic analysis of mixed culture samples (dual transcriptomic analysis), total RNA sequences were mapped to the complete genome of FA350^T^ using the method reported by Westermann et al. [59]. Before downstream processing, trimmomatic was used to clean the reads to remove adaptor sequences and leading and trailing bases with quality thresholds below 20, perform sliding window trimming (with parameters 4 : 15), and remove reads less than 36 bp in length. After cleaning, the remaining paired reads were mapped to the respective genomes to calculate expression values. The reference genomes used were *B. sediminis* FA350^T^ (accession: CP030032.1) and *A. marinus* am2 (accession: MSPQ00000000.1). The output fragments per kilobase of transcript per million mapped reads (FPKM) were calculated for further analysis. Significantly upregulated and downregulated genes were defined using a false discovery rate of less than 0.001, a *P* value of < 0.05, and a minimum 1 log2(fold)-change of gene expression.

### Phylogenetic analysis of bradymonabacterial type IV pili

An unrooted, maximum likelihood phylogeny shows relationships between the type IVa, type IVb, and type IVc pili and the archaellum (archaeal flagellum) and the T2SS and T4SS extension ATPases. The protein amino-acid sequences were aligned with mafft and used to estimate a maximum likelihood phylogeny with RAxML under the JTT substitution model with gamma-distributed rate variation. The protein amino-acid sequences of *Bradymonas* were annotated by RAST (Rapid Annotation using Subsystem Technology) [60]. The other protein amino acid sequences were obtained from other research supplementary materials [61].

### Biogeographic distribution database construction

All the 16S rRNA gene sequences analyzed in this paper were downloaded from the European Nucleotide Archive (https://www.ebi.ac.uk/ena, ENA) during or before December 2018. As a result, we collected 1552 samples from 102 projects or studies: 25 from nonsaline lake sediments (NSLS), 275 from marine sediments (MS), 499 from nonsaline soil (NSS), 45 from saline soil (SS), 216 from nonsaline water (NSW), 19 from saline lake sediments (SLS), 466 from seawater (SW), and 7 from saline lake water (SLW) (Table S5).

### Microbial community composition

The raw 16S rRNA gene reads were filtered with UCHIME. Quality filtering, chimera detection, dereplication, clustering into OTUs, and assigning taxonomic information were performed using VSEARCH [62]. The SILVA database Ref_SSU release 132 was used as a reference taxonomic database (https://www.arb-silva.de/). Alpha diversity indices (Shannon, Simpson, Good’s coverage and Ace) detailing the microbial community composition within each sample were calculated using scikit-bio (http://scikit-bio.org/) in Python, and alpha diversity indices (Chao1) were calculated using the package fossil (https://www.rdocumentation.org/packages/fossil) in R. For estimating community dissimilarities, the Bray–Curtis dissimilarity was calculated between 1551 samples (total samples: 1552) by vegan in R based on the relative abundance of order taxonomy level.

### Phylogenetic analyses

Both RAxML [63] and FastTree [64] were employed to construct the Bradymonabacteria phylogenetic tree. Given both the topology of the phylogenetic tree and its good coverage of all Bradymonabacteria lineages, we established the phylogenetic tree using 187 representative Bradymonabacteria 16S rRNA gene sequences, which were all longer than 1200 bp (at 98.5% cutoff). These sequences were aligned using mafft. The Bradymonabacteria subgroup designations were confirmed when one subgroup with > 10 representative sequences was monophyletic by two phylogenetic trees constructed by different programs using the maximum likelihood approach [65]. The environmental type (i.e., saline and nonsaline) of each Bradymonabacteria sequence in the tree was collected from GenBank. A genome-based phylogeny of bacterial predators and 9 cultured Bradymonabacteria strains was constructed using core genes [66], and trees were constructed using RAxML [63]. All phylogenetic trees were drawn using ggtree [67] in R.

### Quantitative real-time PCR

The environmental DNA samples extracted in the previous step were used for qPCR experiments in order to detect the abundance of bacteria and *Bradymonadales* in each sample. The primer pair composed of 341F (5′-CCTACGGGAGGCAGCAG-3′) and 534R (5′-ATTACCGCGGCTGCTGGCA-3′) was used for quantification of bacteria [22]. A *Bradymonadales*-specific primer set composed of qBRA1295F (5′-CTCAGTWCGGATYGYAGTCTG-3′) and qBRA1420R (5′-GTCACYGACTTCTGGAGCAARYG-3′), which was designed in the present study and generated an amplicon of 148 bases, was used for quantification of *Bradymonadales*. The specificity and coverage test of the primers were described in the Supplementary Materials (Supplementary Methods and Results, Fig. S10 and S11, Tables S8 and S9). Reactions for each sample were carried out in an ABI StepOnePlus thermal cycler under the following conditions: an initial denaturation step at 95 °C for 10 min and then 40 cycles of 15 s at 95 °C and 30 s at 60 °C. The reaction was performed in a total volume of 20 μl, composed of 10 μl 2X Universal SYBR Green Fast qPCR Mix (ABclonal), 0.4 μl of each primer (10 μM), 1 μl of sample, and 8.2 μl of MiliQ water. The Plasmid DNA Standard was constructed by introducing the 16S rDNA gene amplified from *Bradymonas sediminis* FA350^T^ into the pMD18-T Vector (TaKaRa) following the manufacturer’s instructions. The plasmid was isolated and purified using a MiniBEST Plasmid Purification Kit (TaKaRa). DNA copy number was determined by the concentration and relative molecular weight of the Plasmid DNA. For each QPCR assay, the plasmid aliquot was serially diluted to produce concentrations ranging from 10^9^ to 10^3^ DNA copies/μl to generate calibration curves. Each sample was measured in triplicate, and negative controls (no template NTC) were included.

## Supplementary information

**Additional file 1: Figure S1.** The General features of bacterial predators. **Figure S2.** General gene expression profiles of *Bradymonas sediminis* FA350 during mix-culturing with prey *Algoriphagus marines* am2. **Figure S3.** Gene expression profiles of *Bradymonas sediminis* FA350 during mix-culturing with prey *Algoriphagus marines* am2. **Figure S4.** Phylogenetic analysis of secretion system machinery reveals a distinct tad type IVb subtype of type IV pili. **Figure S5.** The alpha diversity in 1,552 samples. **Figure S6.** Principal component analysis of different samples associated with saline status. **Figure S7.** Global distribution of *Myxococcales* and *Bdellovibrionales* in eight different biotopes from 1,552 samples. **Figure S8.** The relative abundance of *Bradymonadales*, *Myxococcales*, and *Bdellovibrionale.* **Figure S9.** Cell abundance and relative abundance of gene copy number of *Bradymonadales* from solar saltern sediments. **Figure S10.** Specificity test of the Quantitative real-time PCR primers. **Figure S11.** Quantitative real-time PCR amplification detection and the standard curve.

**Additional file 2: Table S1.** Detailed information of organisms on the phylogenetic tree

**Additional file 3: Table S2.** Detailed genes information for figure 3.

**Additional file 4: Table S3.** Detailed genes information for figure 4.

**Additional file 5: Table S4.** Detailed key genes expression changed during predation.

**Additional file 6: Table S5.** Detailed information of 16S rRNA genes amplicon samples used in this study.

**Additional file 7: Table S6.** Detailed information of the representative 16S rRNA gene sequences.

**Additional file 8: Table S7.** Probes designed and optimized in this study.

**Additional file 9: Table S8.** Specificity and coverage of primers qBRA1295F and qBRA1420R using the SILVA database SSU r138 Ref NR.

## Data Availability

The genomes of cultured bradymonabacteral isolates have been deposited in the NCBI database under GenBank accession numbers CP042467.1 (*Bradymonadales* strain V1718), CP042468.1 (*Bradymonadales* strain YN101), VOPX00000000.1 (*Bradymonadales* strain TMQ1), VOSL00000000.1 (*Bradymonadales* strain TMQ2), QRGZ00000000.1 (*Bradymonadales* strain TMQ3), VOSM00000000.1 (*Bradymonadales* strain TMQ4), CP030032.1 (*Bradymonas sediminis* FA350^T^), QHKO00000000.1 (*Lujinxingia litorali* B210^T^), and SADD00000000.1 (*Lujinxingia sediminis* SEH01^T^). The genomes of uncultured Bradymonabacteria have been deposited in the NCBI database under GenBank accession numbers PWKZ00000000.1 (*Bradymonadales* bin CSSed10_215), PWTN00000000.1 (*Bradymonadales* bin CSSed11_191), PXAJ00000000.1 (*Bradymonadales* bin T3Sed10_204), and PWZZ00000000.1 (*Bradymonadales* bin T3Sed10_190). The 16S rRNA gene data sets of Gaodao salterns have been deposited in the Sequence Read Archive under accession number SRP217756 for all the samples. The transcriptome sequences for predation of FA350^T^ have been deposited in the NCBI database under accession numbers PRJNA559243 and PRJNA559253. All Bradymonabacteral isolates have been deposited at the Shandong Infrastructure of Marine Microbial Resources hosted by the Laboratory of Marine Microbiology at Shandong University (http://www.sdum.wh.sdu.edu.cn/search.html?itemId=14). All Bradymonabacteral isolates are available upon request. The bash, R, and python scripts for this study are available on the GitHub: https://github.com/2015qyliang/BradymonabacteriaAnalysis.
