# Supplementary material for: Bradymonabacteria, a novel bacterial predator group with versatile survival strategies in saline environments
Source: Microbiome. 2020 Aug 31;8:126. doi: 10.1186/s40168-020-00902-0 (PMC7460792; doi:10.1186/s40168-020-00902-0)
Supplement: Supplementary file 2 — Additional file 1: Figure S1. The General features of bacterial predators. Figure S2. General gene expression profiles of Bradymonas sediminis FA350 during mix-culturing with prey Algoriphagus marines am2. Figure S3. Gene expression profiles of Bradymonas sediminis FA350 during mix-culturing with prey Algoriphagus marines am2. Figure S4. Phylogenetic analysis of secretion system machinery reveals a distinct tad type IVb subtype of type IV pili. Figure S5. The alpha diversity in 1,552 samples. Figure S6. Principal component analysis of different samples associated with saline status. Figure S7. Global distribution of Myxococcales and Bdellovibrionales in eight different biotopes from 1,552 samples. Figure S8. The relative abundance of Bradymonadales, Myxococcales, and Bdellovibrionale. Figure S9. Cell abundance and relative abundance of gene copy number of Bradymonadales from solar saltern sediments. Figure S10. Specificity test of the Quantitative real-time PCR primers. Figure S11. Quantitative real-time PCR amplification detection and the standard curve. [file 40168_2020_902_MOESM1_ESM.docx]

**Supplementary Materials**

**Bradymonabacteria, a novel bacterial predator group with versatile survival strategies in saline environments**

Da-Shuai Mu^1,2^, Shuo Wang^2^, Qi-Yun Liang^2^, Zhao-Zhong Du^2^, Renmao Tian^3^, Yang Ouyang^3^, Xin-Peng Wang^2^, Aifen Zhou^3^, Ya Gong^1,2^, Guan-Jun Chen^1,2^, Joy Van Nostrand^3^, Yunfeng Yang^4^, Jizhong Zhou^3,4^, Zong-Jun Du^1,2*^

^1^ State Key Laboratory of Microbial Technology, Institute of Microbial Technology, Shandong University, Qingdao, 266237, China

^2^ Marine College, Shandong University, Weihai, 264209, People’s Republic of China

^3^ Institute for Environmental Genomics, University of Oklahoma, Norman, Oklahoma, USA.

^4^ State Key Joint Laboratory of Environment Simulation and Pollution Control, School of Environment, Tsinghua University, Beijing, China.

*Address correspondence to:

Zong-Jun Du, E-mail: [duzongjun@sdu.edu.cn](mailto:duzongjun@sdu.edu.cn); Tel.: +86-0631-5688303. Mailing address: State Key Laboratory of Microbial Technology, Shandong University, No. 72, Jimo Binhai Road, Jimo, Qingdao 266237, China

This file includes:

**Methods**

**Results**

**Figs. S1 to S11**

**References**

**Supplementary Methods**

**Multi-pond salterns sampling**

Samples were obtained from Gaodao multi-pond saltern (Weihai, China, 36° 59′ 38″ N 122° 1′ 45″ E, Weihai, China) on 1 May 2019 and included samples from five ponds with salinity of 45, 80, 125, 175 and 265‰. Sediment and water samples were taken from each pond. For every pond, salinity, water temperature and pH values were measured *in situ*. Each pond was sampled in three random locations by collecting sediment samples for DNA extraction and FISH detection.

**DNA extraction and sequencing for Multi-pond salterns samples**

Sediment (0.8 g) was taken from each sample for DNA extraction using a FastDNA Spin Kit for soil (MP Biomedical, France) following the manufacturer's instructions. The extracted DNA was divided into two parts, one for high-throughput sequencing and the other for subsequent qPCR testing. The prime set composed of 515F (5′-GTGCCAGCMGCCGCGG-3′) and 907R (5′-CCGTCAATTCMTTTRAGTTT-3′) targeting the bacterial V4–V5 region were selected for the microbial community structure analysis. Sequencing was carried out on a MiSeq platform at the Majorbio Bio-Pharm Technology Co., Ltd. (Shanghai, China).

**FISH probe design**

Design of the fluorescence in situ hybridization (FISH) probe for *Bradymonadales* was performed using the ARB software package [1] based on the SILVA database SSU Ref NR99, release 132 [2]. Before designing the probe, we manually added the 16S rRNA sequences of seven strains of *Bradymonadales* that had been isolated and cultured by us and were not available in the SILVA database. Further information on the resultant probes with their corresponding competitor and helper oligonucleotides is available in Table S7. The 5′ ends of the probes designed for *Bradymonadales* were labeled with dye Cy3. The EUB mix probes which targeting most bacteria [3] were labeled with fluorescein isothiocyanate (FITC). The NON-EUB nonsense probe was used as a negative control for hybridization.

**Fixation of sample and FISH**

The sediment samples were fixed in 4% (w/v) paraformaldehyde for 12 hours at 4 °C and washed twice with 1×PBS. After fixation, the samples were stored in 1× PBS/EtOH at −20 °C. Samples (200 μL) were resuspend in 2 mL 1× PBS with 0.01 M Na-pyrophosphate and sonicated on ice at low intensity. Then, 1 mL of Nycodenz (AXIS-SHIELD PoC, Oslo, Norway) solution (1.3g mL^-1^) was carefully added in the bottom of the tube and the sample was centrifuged in a swing-bucket rotor (14,000g for 90 min at 4 °C). After centrifugation, the Nycodenz layer and the upper layer solution were collected onto 25mm polycarbonate filters with a 0.2 µm pore size (GTTP, Millipore, Eschborn, Germany). Permeabilization and FISH on filters was performed as described by Katrin Zwirglmaier [4]. Microscopy was performed with an Axioscope A1 epifluorescence microscope (Carl Zeiss, Oberkochen, Germany).

**Quantitative real-time PCR primer design and test**

The primers were designed based on the 16S rRNA sequences of cultivated and uncultured *Bradymonadales* sequences in the SILVA database. The test Prime function in the SILVA website (<https://www.arb-silva.de/search/testprime/>) was used to test the specificity and coverage of the primers. To verify the specificity of the PCR primers, 9 *Bradymonadales* strains and 5 non-target strains were cultured (Fig. S1), and the genomic DNA of the strains was extracted and purified using a TaKaRa MiniBEST Bacterial Genomic DNA Extraction kit (Takara Biomedical Technology Beijing Co., Ltd.). In addition, genomic DNA from an environmental Gaodao saltern sediment sample was extracted using a FastDNA Spin kit for soil (MP Biomedical, France). Genomic DNA (10 ng) was used for PCR verification with the following thermocycling conditions: 94 ℃ for 6 min followed by 30 cycles of 94 ℃ for 45 s, 60 ℃ for 45 s and 72℃ for 10 s, with a final incubation at 72℃ for 5 min. PCR amplicons obtained using DNA from the Gaodao saltern sediment sample WD9 were purified using a PCR product purification kit (Takara) and cloned into the vector pMD18-T (Takara). Recombinant plasmids were reproduced in *Escherichia coli* DH5α cells. Selection, amplification and sequencing of positive clones was performed, after which the sequencing results of the clones were BLASTed against the NCBI database to verify the specificity of amplification.

**Supplementary Results**

**General features of the genomes of *Bradymonadales***

Almost all of the 13 genomes appear to have the potential for a complete electron-transport chain, glycolysis, folate C1 metabolism, and tricarboxylic acid (TCA) cycle (Fig. 2).

Certain members of *Bradymonadales* tend to utilize starch to generate glucose for glycolysis. However, some key genes involved in utilizing carbohydrates such as disaccharides hydrolase and N-acetylglucosamine-6-phosphate deacetylase (NagA) are missing, providing genomic evidence for experimental evidence of a poor ability utilize carbohydrates [5, 6]. Nitrite reductase could be responsible for conversion of nitrite to ammonia that is then used for the biosynthesis of amino acids in *Bradymonadales*, suggesting that the strains could be a type of dissimilatory nitrate reduction to ammonium (DNRA) bacteria [7] and use inorganic nitrogen. Several strains have an incomplete respiratory nitrate reduction, and all the strains lacked nitric oxide reductase, resulting in the generation of nitric oxide, instead of nitrous oxide and nitrogen (Fig. 2). Most Bradymonabacteria possess hemoglobins (nitric oxide dioxygenase) and NnrS (involved in response to NO) to protect Bradymonabacteria from nitric oxide and nitrosative stresses [8]. Unlike most other orders in *Deltaproteobacteria*, the sulfate-reducing pathway is absent in all of the Bradymonabacteria. Most genomes harbored genes encoding sulfatases, suggesting that utilization of organosulfur compounds is a common feature among *Bradymonadales.*

All genomes possessed several genes encoding sodium symporters and Na^+^/H^+^ antiporters, suggesting these bacteria have adapted to a saline environment. Almost all genomes contained outer membrane protein A precursor (OmpA), important for adapting to osmotic pressure under hyperosmotic stress. These results further support the notion that *Bradymonadales* has a preference for saline environments.

**Quantitative real-time PCR primer specificity test**

The designed qPCR primer pairs and their coverage and specificity for *Bradymonadales* are shown in Table S8. Compared with the pure cultures of non-target strains, the conventional PCR results obtained with pure *Bradymonadales* strains showed that an approximately 148-bp product could be amplified from all 9 *Bradymonadales* strains and environmental samples, while the results for the 5 non-target bacteria were negative (Fig. S10). Furthermore, based on the NCBI BLAST analysis results, all 27 cloned sequences obtained from the environmental sample WD9, a sediment sample from the Gaodao saltern, belong to the *Bradymonadales* group, indicating the high specificity of primer amplification in environmental samples (Table S9). The qPCR experiment with the designed primers yielded an acceptable standard curve (R^2^=0.99), and the dissolution curve of qPCR amplification was a single peak (Fig. S11), suggesting the specific amplification of the designed primers during qPCR.


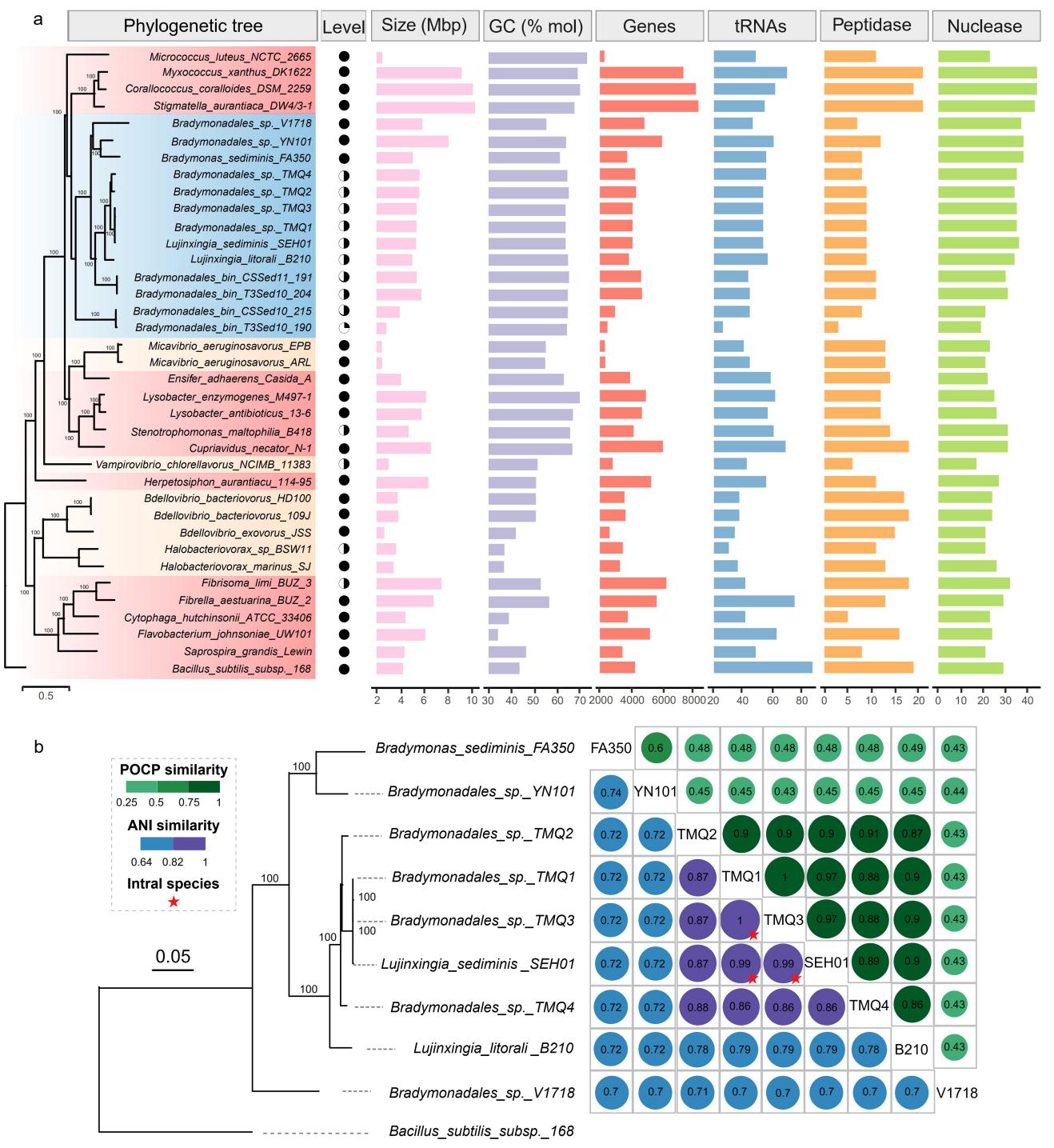


**Figure S1. The General features of bacterial predators.**

**a,** RaxMl tree was built by using core gene list. Trees in red background indicate the known facultative predators, trees in yellow background indicate the known obligate predators, and trees in blue background indicate Bradymonabacteria. Circles in Level Column indicate the assembly level, black solid circle indicates complete genome, half solid circle indicates scaffold assembly. Bar graph indicates some general features about bacterial genome. **b,** ANI and POCP analysis of 9 cultured strains of *Bradymonadales***.** Red star indicates different strains belong to the same species. RaxMl tree was built by using core gene list.


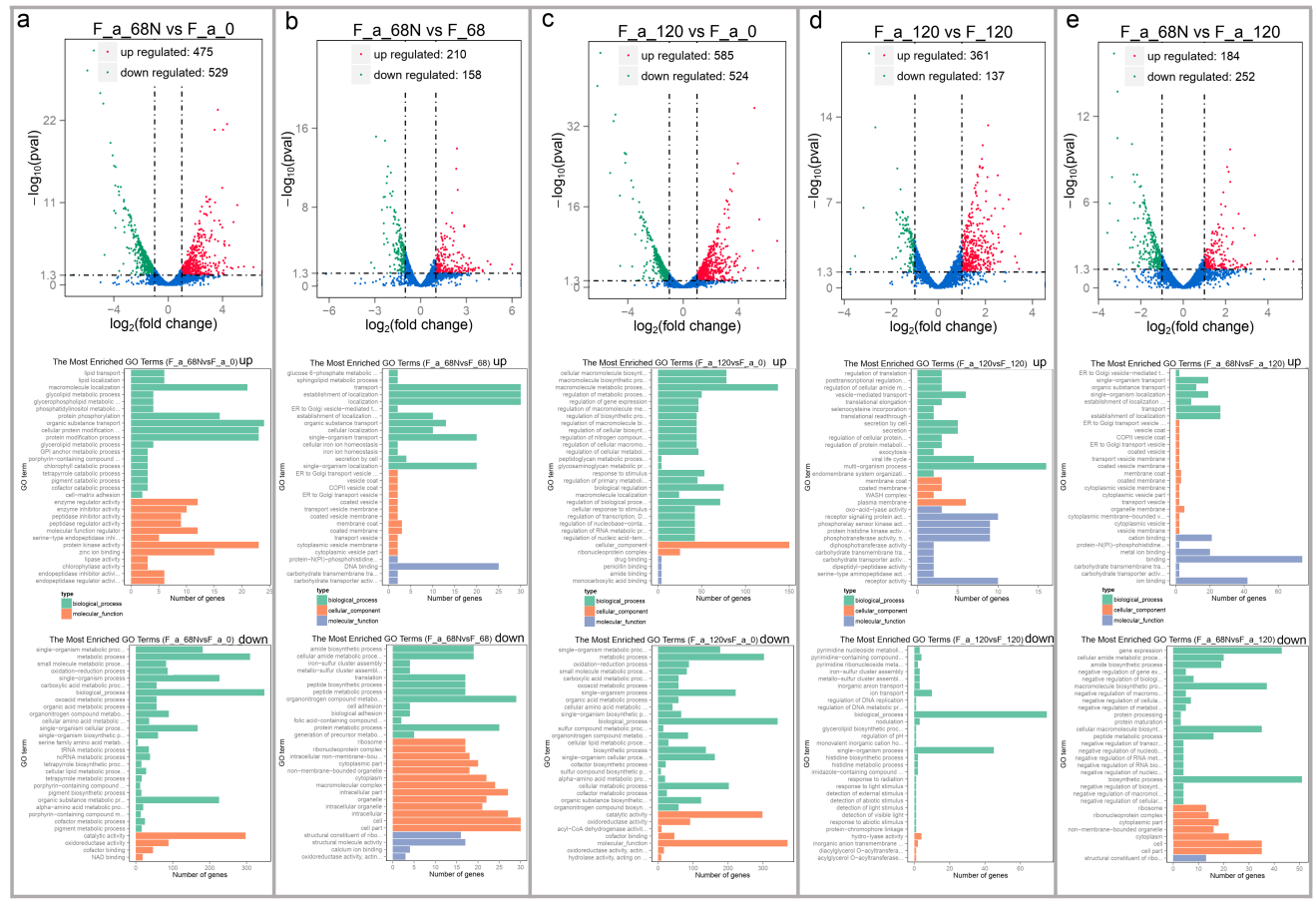


**Figure S2. General gene expression profiles of *Bradymonas sediminis* FA350 during mix-culturing with prey *Algoriphagus marines* am2.**

Displayed are the volcano plots and enriched gene clusters of up or down regulated expression showing pairwise comparisons of gene expression between the different treatments. **a,** indicate prey-predator mix-culture 68 hrs/0 h; **b,** indicate prey-predator mix-culture 68 hrs/ predator pure culture 68 hrs; **c,** indicate prey-predator mix-culture 120 hrs/0 h; **d,** indicate prey-predator mix-culture 120 hrs/ predator pure culture 120 hrs; **e,** indicate prey-predator mix-culture 68 hrs/mix-culture 120 hrs. n=3, p<0.05.


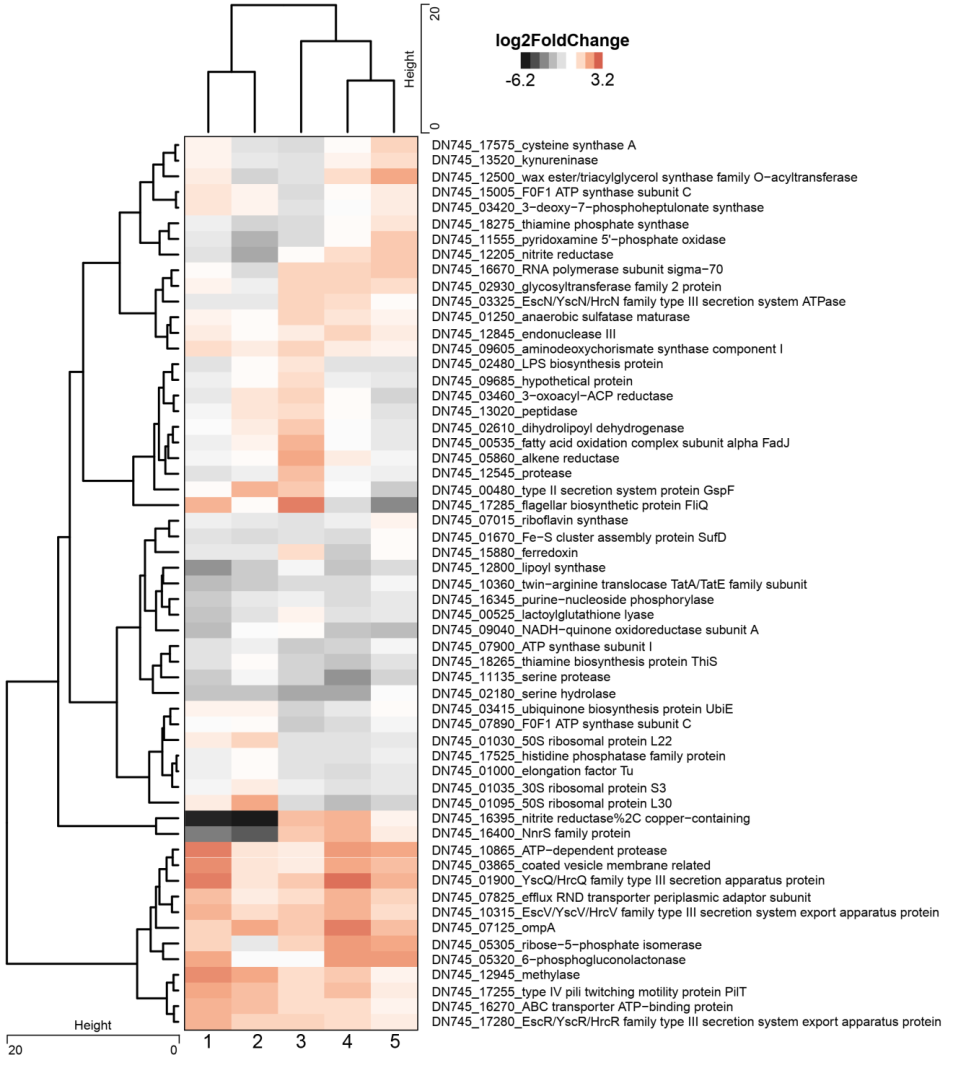


**Figure S3. Gene expression profiles of *Bradymonas sediminis* FA350 during mix-culturing with prey *Algoriphagus marines* am2.**

Displayed are the relative expression levels of each gene. The numbers 1, 2, 3, 4, and 5 indicate prey-predator mix-culture 68 hrs/0 h, mix-culture 120 hrs/0 h, mix-culture 120 hrs/pure culture 120 hrs, mix-culture 68 hrs/pure culture 68 hrs, and mix-culture 68 hrs/mix-culture 120 hrs, respectively. The color indicates the mean of log2 fold change of expression (Table S4). Color indications are red and orange for increased expression, black and grey for decreased expression. n=3, p<0.05.


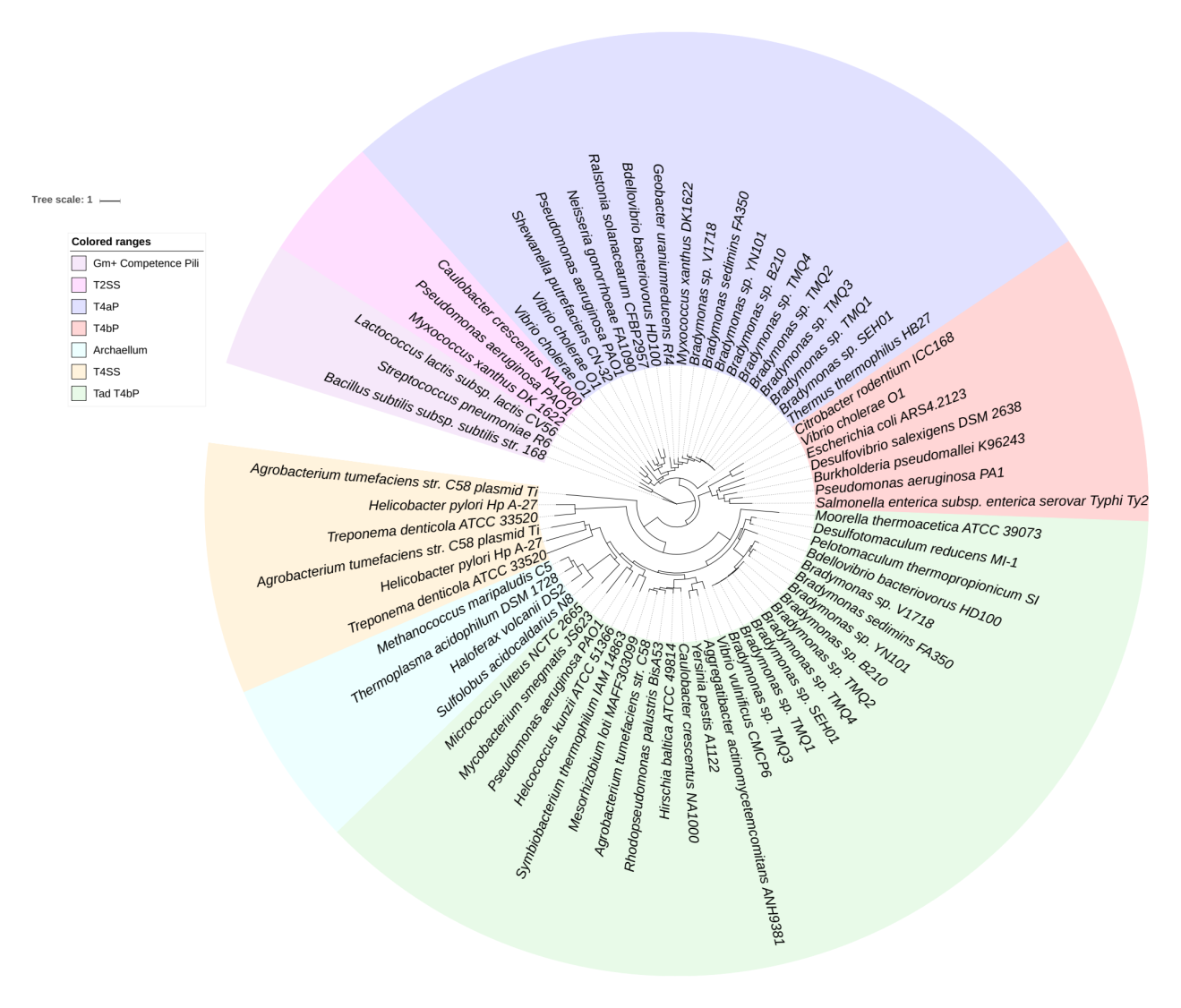


**Figure S4. Phylogenetic analysis of secretion system machinery reveals a distinct tad type IVb subtype of type IV pili.**

An unrooted, maximum likelihood phylogeny shows relationships between type IVa, type IVb, tad type IVb pili, archaellum (archaeal flagellum), T2SS, and T4SS extension ATPases. An ancestral duplication event in the type IVb tad platform proteins indicate there were two platform proteins for these pili in Bradymonabacteria.


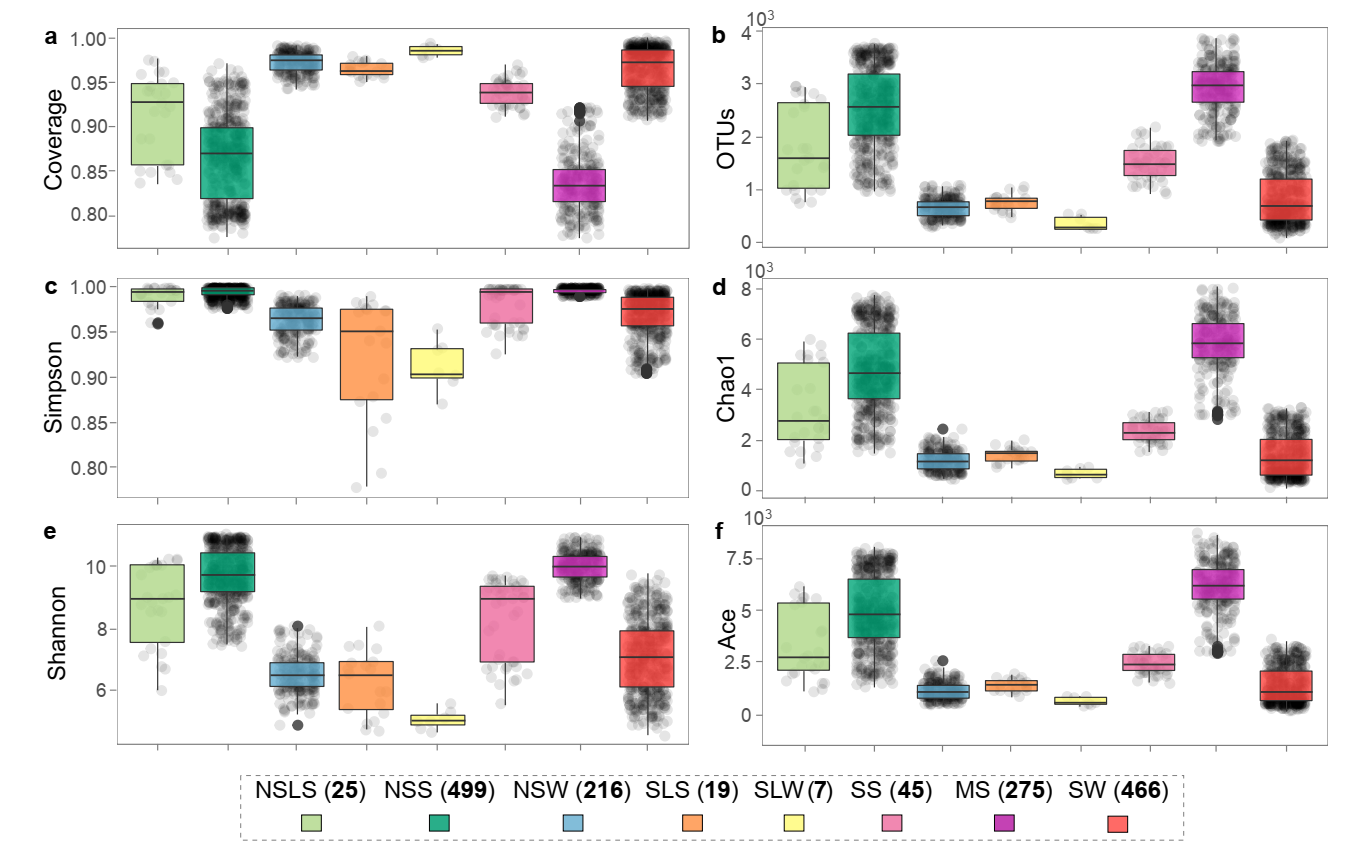


**Figure S5. The alpha diversity in 1,552 samples.**

Within-community (alpha) diversity, measured as number of observed sequences (richness), in 1,552 biologically independent samples among eight different biotopes, with boxplots showing median inter-quartile range (IQR), and 1.5 × IQR (with outliers)**.** Bold numbers represents the number of samples in each biotopes. NSLS, non-saline lake sediments; NSS, non-saline soil; NSW, non-saline water; SW, sea water; MS, marine sediments; SS, saline soil; SLS, saline lake sediments; SLW, saline lake water.


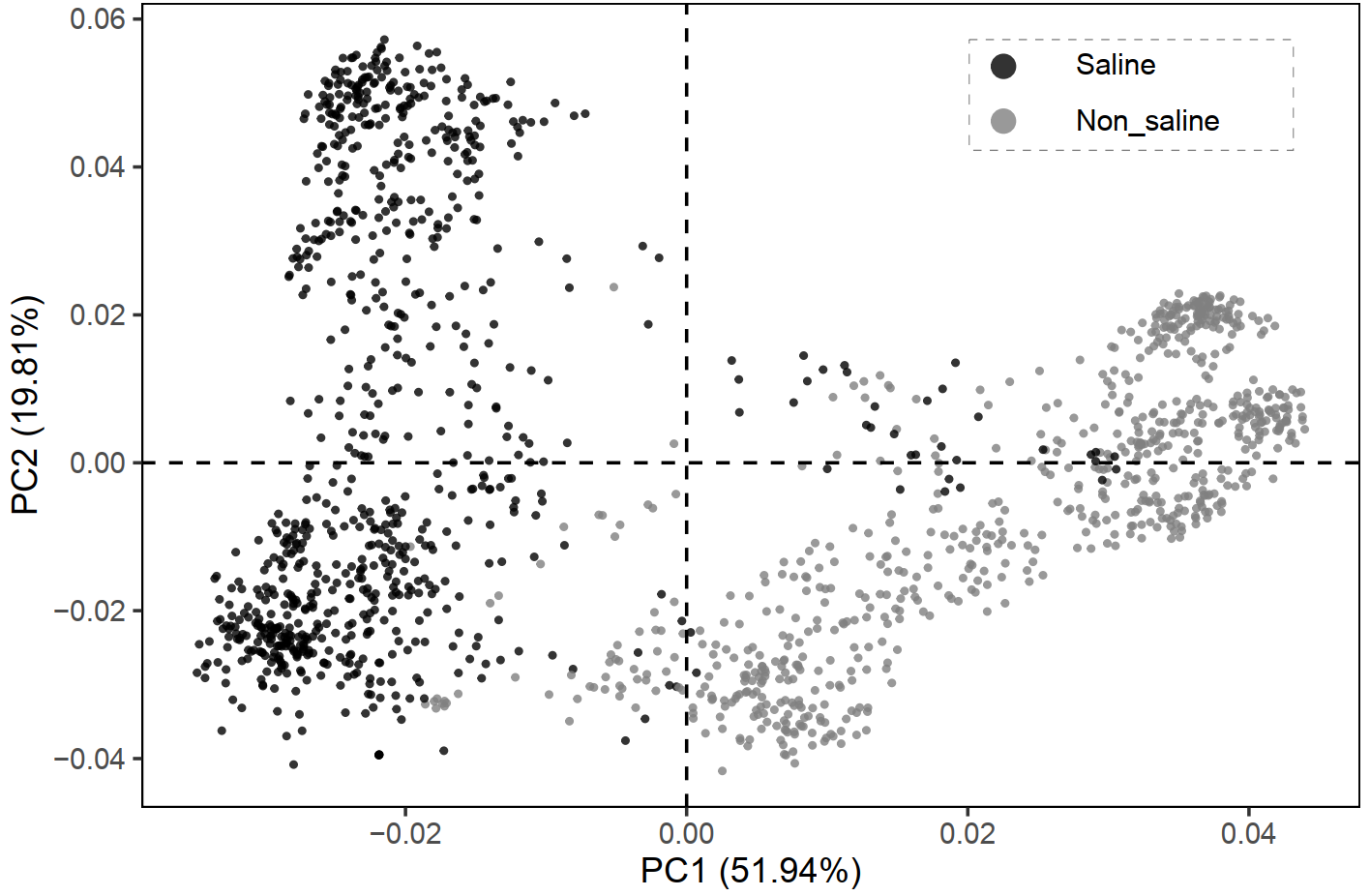


**Figure S6. Principal component analysis of different samples associated with saline status.**

Beta-diversity of all samples among two different types of habitats: principal component analysis (PCA) of Bray-Curtis dissimilarity matrix, PC1 versus PC2. Clustering of all samples could be largely explained by the type of habitats (saline: black, non-saline: gray). Bold numbers represents the number of samples in different saline status.


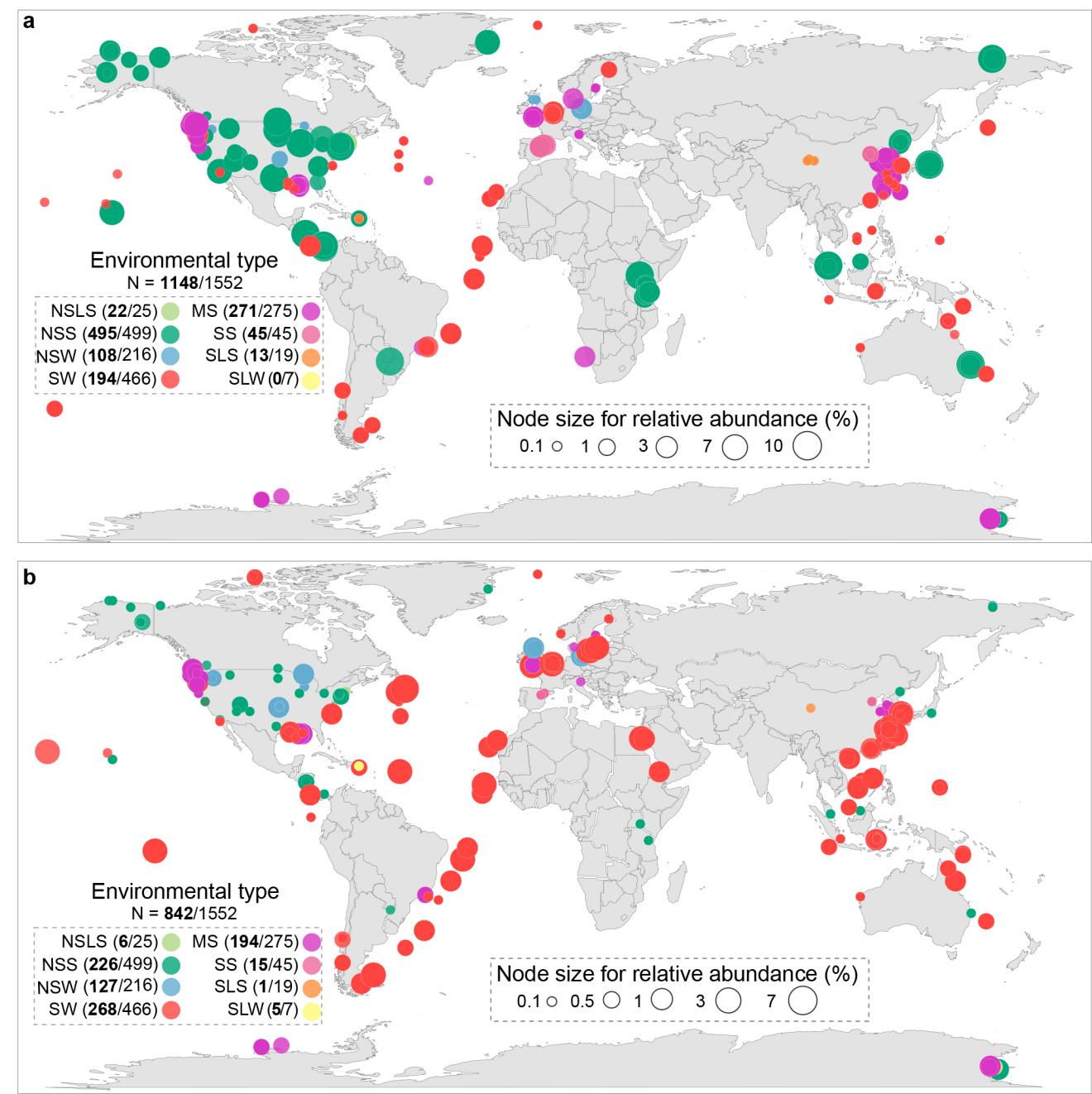


**Figure S7. Global distribution of *Myxococcales* and *Bdellovibrionales* in eight different biotopes from 1,552 samples.**

**a**, Global abundance of *Myxococcales*. **b,** Global abundance of *Bdellovibrionales*. The abundance of 16S rRNA gene sequences of *Myxococcales* or *Bdellovibrionales* is relative to total prokaryotic sequences in the selected samples. Each node represents one sample. Node color indicates the type of biotopes, and node size represents the relative abundance in the corresponding samples. Bold numbers represents the number of samples, which detected *Myxococcales* and *Bdellovibrionales,* respectively.


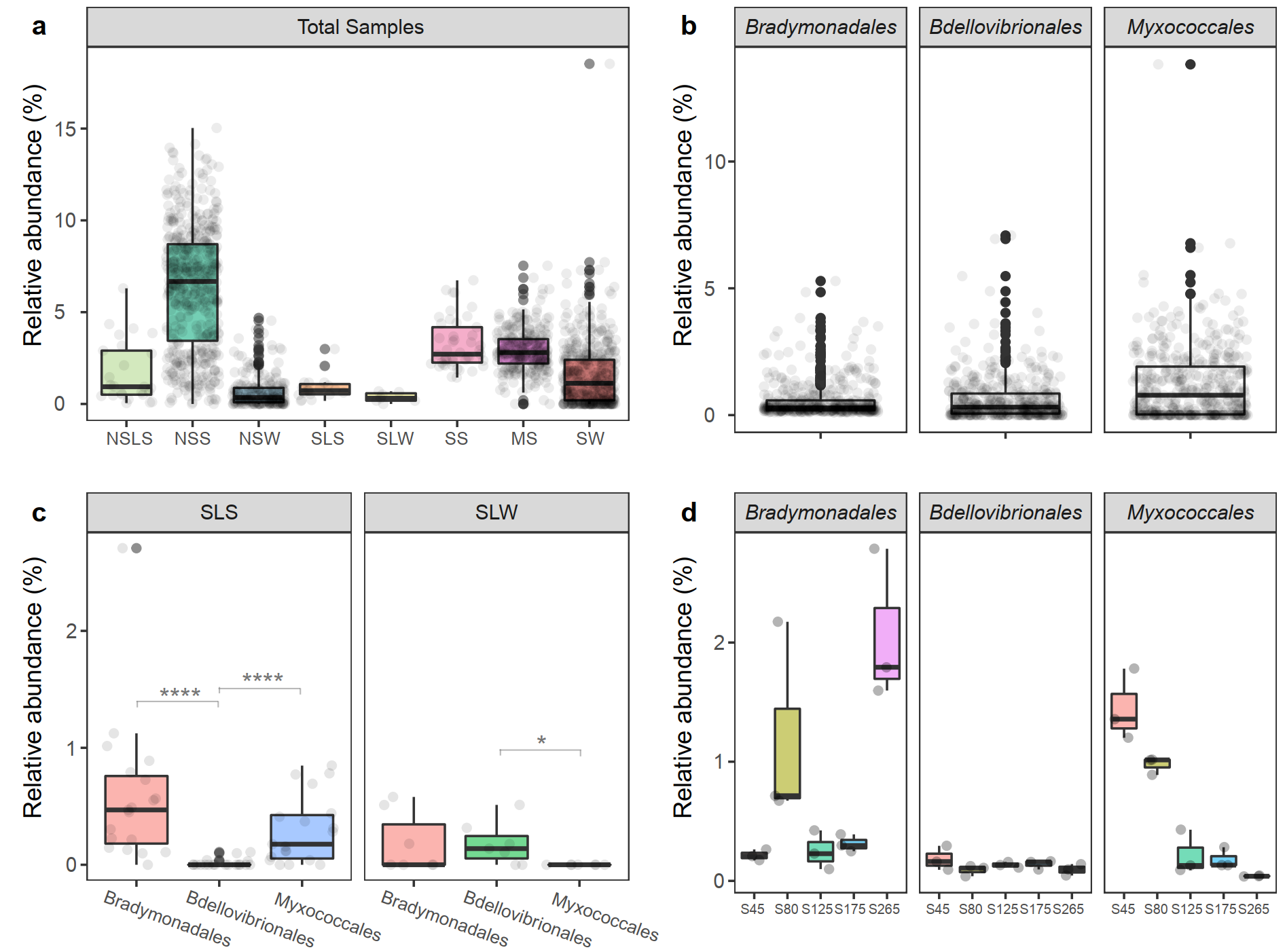


**Figure S8. The relative abundance of *Bradymonadales*, *Myxococcales*, and *Bdellovibrionale.***

**a,** Relative abundance of bacterial predators (sum of *Bradymonadales*, *Myxococcales*, and *Bdellovibrionales*) in all 1,552 samples. **b,** Relative abundance (> 0.1%) of *Bradymonadales*, *Myxococcales*, and *Bdellovibrionales* in all samples which detected each predator. **c,** Relative abundance of *Bradymonadales*, *Myxococcales*, and *Bdellovibrionales* in saline lake sediment (SLS) and saline lake water (SLW) samples. The significant differences among different predator groups was assessed by Kruskal−Wallis test. * P<0.05; **** P<0.0001. **d,** Relative abundance of *Bradymonadales*, *Myxococcales*, and *Bdellovibrionales* in Gaodao multi-pond saltern samples in this study. n=3, * P<0.05.


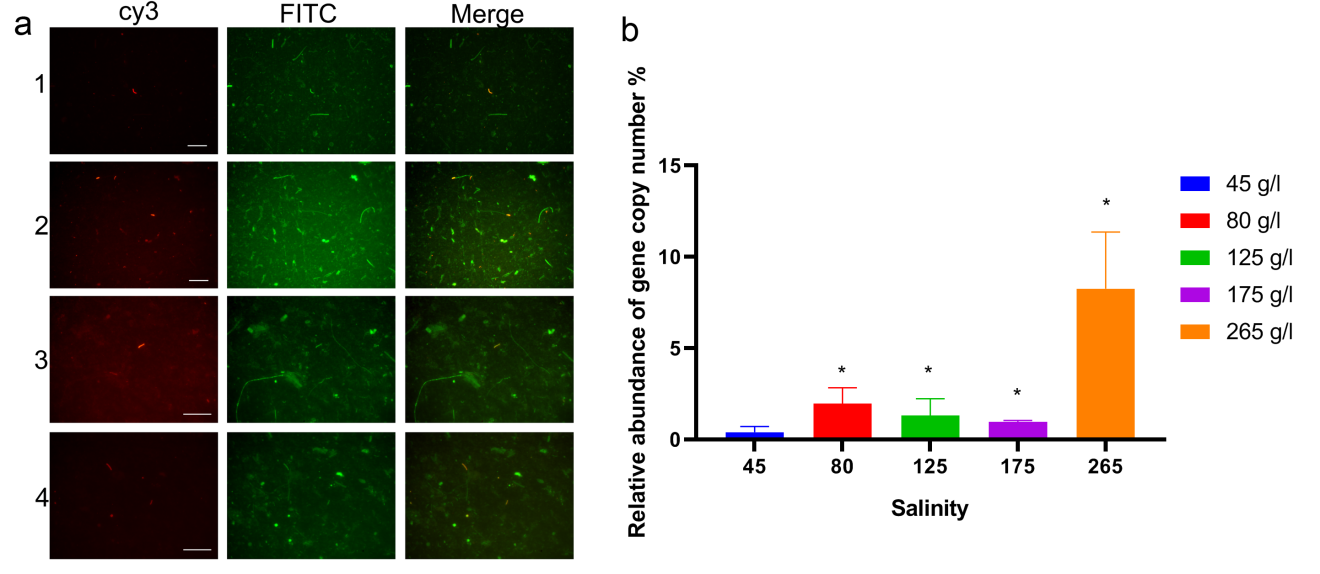


**Figure S9. Cell abundance and relative abundance of gene copy number of *Bradymonadales* from solar saltern sediments.**

**a,** Epifluorescent image after FISH with probe BRA1425 and BRA835 targeting some rod- members of the *Bradymonadales* (labeled with cy3, in red fluorescence), with EUB338 targeting most bacteria (labeled with fluorescein isothiocyanate FITC, in green fluorescence). Merge indicate the red fluorescence and green fluorescence merged together, and *Bradymonadales* could be shown in orange color. 1, samples from the solar saltern with salinity 45 g/L; 2, samples from the solar saltern with salinity 80 g/L; 3, samples from the solar saltern with salinity 125 g/L; 4, samples from the solar saltern with salinity 175 g/L. Scale bar refers to 10 µm. **b,** Relative abundance of *Bradymonadales* compared with bacteria by 16s rRNA gene copy number.


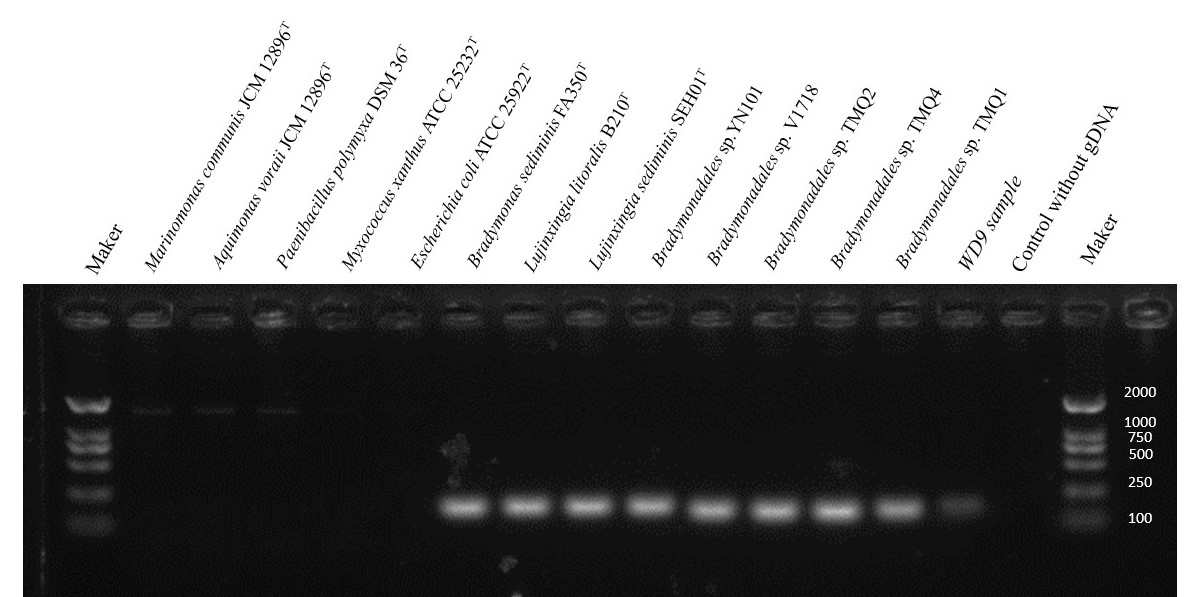


**Figure S10. Specificity test of the Quantitative real-time PCR primers.**

Verification of prime qBRA1295F and qBRA1420R in PCR runs, different channels were different DNA samples, Marker is DL2000.


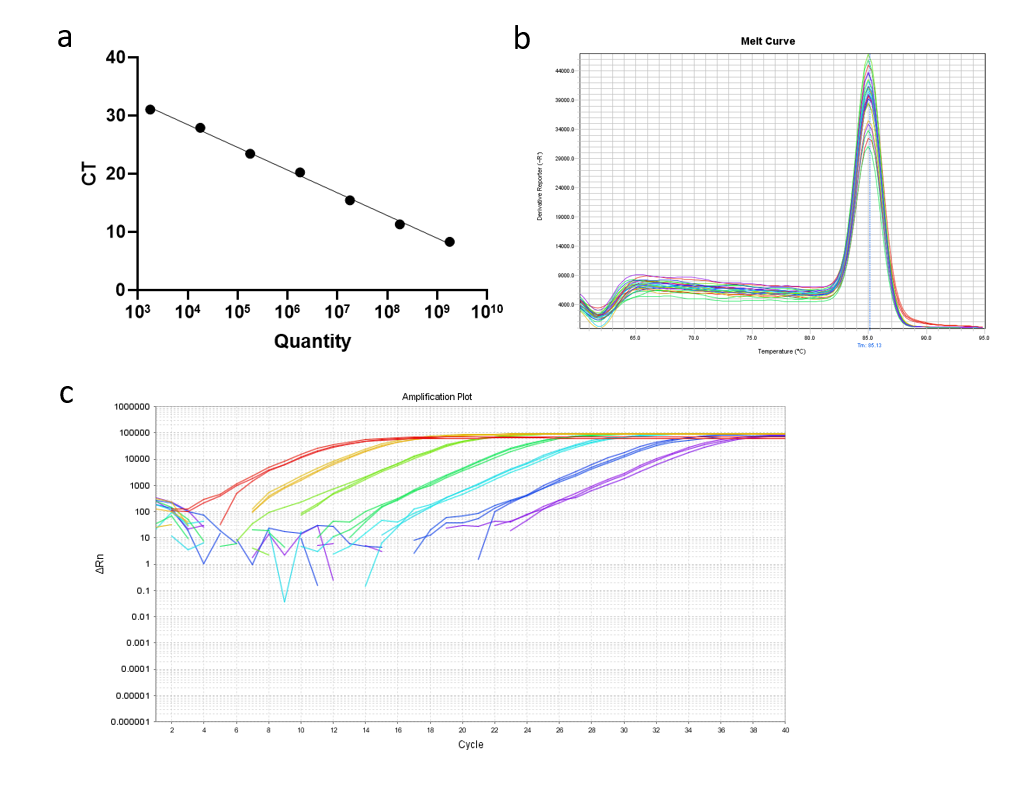


**Figure S11. Quantitative real-time PCR amplification detection and the standard curve.**

**a,** Standard curve; **b,** melting curve; **c,** fluorescence amplification plot of 10-fold series dilutions of plasmid of qPCR environment by primers qBRA1295F and qBRA1420R.

**Reference:**

1. Ludwig W, Strunk O, Westram R, Richter L, Meier H, Yadhukumar, et al. ARB: a software environment for sequence data. Nucleic Acids Research. 2004;32(4):1363-71.

2. Quast C, Pruesse E, Yilmaz P, Gerken J, Schweer T, Yarza P, et al. The SILVA ribosomal RNA gene database project: improved data processing and web-based tools. Nucleic Acids Research. 2013;41(D1):D590-D6.

3. Daims H, Bruhl A, Amann R, Schleifer KH, Wagner M. The domain-specific probe EUB338 is insufficient for the detection of all Bacteria: Development and evaluation of a more comprehensive probe set. Syst Appl Microbiol. 1999;22(3):434-44.

4. Zwirglmaier K. Detection of prokaryotic cells with fluorescence in situ hybridization. Methods Mol Biol. 2010;659:349-62.

5. Wang ZJ, Liu QQ, Zhao LH, Du ZJ, Chen GJ. Bradymonas sediminis gen. nov., sp. nov., isolated from coastal sediment, and description of Bradymonadaceae fam. nov. and Bradymonadales ord. nov. Int J Syst Evol Microbiol. 2015;65(5):1542-9.

6. Guo LY, Li CM, Wang S, Mu DS, Du ZJ. Lujinxingia litoralis gen. nov., sp. nov. and Lujinxingia sediminis sp. nov., two new representatives in the order Bradymonadales. Int J Syst Evol Microbiol. 2019; doi: 10.1099/ijsem.0.003556.

7. Kraft B, Strous M, Tegetmeyer HE. Microbial nitrate respiration - Genes, enzymes and environmental distribution. Journal of Biotechnology. 2011;155(1):104-17.

8. Stern AM, Hay AJ, Liu Z, Desland FA, Zhang J, Zhong ZT, et al. The NorR Regulon Is Critical for Vibrio cholerae Resistance to Nitric Oxide and Sustained Colonization of the Intestines. Mbio. 2012;3(2).

9. Ventosa A, Fernandez AB, Leon MJ, Sanchez-Porro C, Rodriguez-Valera F. The Santa Pola saltern as a model for studying the microbiota of hypersaline environments. Extremophiles. 2014;18(5):811-24.
