# Supplementary material for: Bradymonabacteria, a novel bacterial predator group with versatile survival strategies in saline environments
Source: Microbiome. 2020 Aug 31;8:126. doi: 10.1186/s40168-020-00902-0 (PMC7460792; doi:10.1186/s40168-020-00902-0)
Supplement: Supplementary file 9 — Additional file 8: Table S7. Probes designed and optimized in this study. [file 40168_2020_902_MOESM8_ESM.docx]

**Table S8** Specificity and coverage of primers qBRA1295F and qBRA1420R using the SILVA database SSU r138 Ref NR

| **Primer** | **Sequence (5’- 3’)** | **Coverage of *Bradymonadales*** | **Non-target hits** | **Amplicon length (bp)** |
| --- | --- | --- | --- | --- |
| qBRA1295F | CTCAGTWCGGATYGYAGTCTG | 66/235（28%） | 6 | 148 |
| qBRA1420R | GTCACYGACTTCTGGAGCAARYG |  |  |  |
