# Supplementary material for: Bradymonabacteria, a novel bacterial predator group with versatile survival strategies in saline environments
Source: Microbiome. 2020 Aug 31;8:126. doi: 10.1186/s40168-020-00902-0 (PMC7460792; doi:10.1186/s40168-020-00902-0)
Supplement: Supplementary file 10 — Additional file 9: Table S8. Specificity and coverage of primers qBRA1295F and qBRA1420R using the SILVA database SSU r138 Ref NR. [file 40168_2020_902_MOESM9_ESM.docx]

**Table S9.** NCBI BLAST search results of clones

| Clone name | NCBI Sequence Match Species | Max Identity（%） |
| --- | --- | --- |
| BRA1 | *Bradymonadales* sp. YN101 | 99.3 |
| BRA2 | *Lujinxingia litoralis* B210^T^ | 98.0 |
| BRA4 | *Bradymonas sediminis* FA350^T^ | 98.6 |
| BRA5 | *Bradymonas sediminis* FA350^T^ | 98.6 |
| BRA7 | *Lujinxingia litoralis* B210^T^ | 97.3 |
| BRA10 | *Lujinxingia litoralis* B210^T^ | 98.0 |
| BRA13 | *Lujinxingia litoralis* B210^T^ | 97.3 |
| BRA14 | *Lujinxingia litoralis* B210^T^ | 98.6 |
| BRA15 | *Lujinxingia litoralis* B210^T^ | 95.9 |
| BRA16 | *Bradymonas sediminis* FA350^T^ | 98.6 |
| BRA17 | *Bradymonas sediminis* FA350^T^ | 97.3 |
| BRA19 | *Lujinxingia litoralis* B210^T^ | 98.0 |
| BRA20 | *Lujinxingia litoralis* B210^T^ | 98.6 |
| BRA21 | *Bradymonadales* sp. YN101 | 98.6 |
| BRA24 | *Bradymonas sediminis* FA350^T^ | 98.0 |
| BRA25 | *Bradymonadales* sp. YN101 | 100.0 |
| BRA26 | *Bradymonas sediminis* FA350^T^ | 98.0 |
| BRA28 | *Bradymonas sediminis* FA350^T^ | 99.3 |
| BRA29 | *Bradymonas sediminis* FA350^T^ | 98.0 |
| BRA33 | *Lujinxingia litoralis* B210^T^ | 98.0 |
| BRA34 | *Bradymonas sediminis* FA350^T^ | 98.6 |
| BRA40 | *Lujinxingia litoralis* B210^T^ | 98.6 |
| BRA42 | *Bradymonas sediminis* FA350^T^ | 96.6 |
| BRA44 | *Bradymonadales* sp. YN101 | 98.0 |
| BRA45 | *Bradymonas sediminis* FA350^T^ | 97.3 |
| BRA48 | *Lujinxingia litoralis* B210^T^ | 98.0 |
| BRA50 | *Bradymonadales* sp. YN101 | 98.6 |
